# Megalochelin, a Tridecapeptide Siderophore from a Talented Streptomycete

**DOI:** 10.1021/acschembio.2c00958

**Published:** 2023-03-15

**Authors:** Kristiina Vind, Cristina Brunati, Matteo Simone, Margherita Sosio, Stefano Donadio, Marianna Iorio

**Affiliations:** † NAICONS Srl, 20139 Milan, Italy; ‡ Host-Microbe Interactomics Group, Wageningen University, 6708 WD Wageningen, The Netherlands

## Abstract

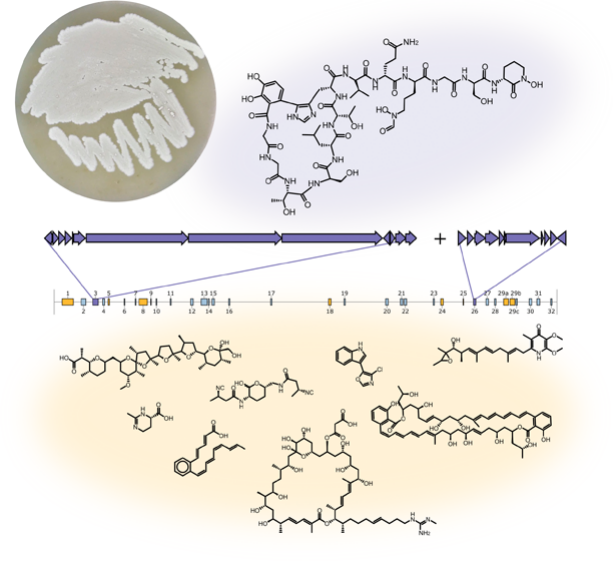

Streptomycetes are bacteria known for their extraordinary biosynthetic capabilities. Herein, we describe the genome and metabolome of a particularly talented strain, Streptomyces ID71268. Its 8.4-Mbp genome harbors 32 bioinformatically predicted biosynthetic gene clusters (BGCs), out of which 10 are expressed under a single experimental condition. In addition to five families of known metabolites with previously assigned BGCs (nigericin, azalomycin F, ectoine, SF2766, and piericidin), we were able to predict BGCs for three additional metabolites: streptochlorin, serpetene, and marinomycin. The strain also produced two families of presumably novel metabolites, one of which was associated with growth inhibitory activity against the human opportunistic pathogen *Acinetobacter baumannii* in an iron-dependent manner. Bioassay-guided fractionation, followed by extensive liquid chromatography–mass spectrometry (LC-MS) and NMR analyses, established that the molecule responsible for the observed antibacterial activity is an unusual tridecapeptide siderophore with a ring-and-tail structure: the heptapeptide ring is formed through a C–C bond between a 2,3-dihydroxybenzoate (DHB) cap on Gly1 and the imidazole moiety of His7, while the hexapeptide tail is sufficient for binding iron. This molecule, named megalochelin, is the largest known siderophore. The megalochelin BGC encodes a 13-module nonribosomal peptide synthetase for the synthesis of the tridecapeptide, and a copper-dependent oxidase, likely responsible for the DHB-imidazole cross-link, whereas the genes for synthesis of the DHB starter unit are apparently specified *in trans* by a different BGC. Our results suggest that prolific producers of specialized metabolites may conceal hidden treasures within a background of known compounds.

The increasing and aging human population is driving the need to find new drug candidates to treat and prevent diseases in humans, animals, and plants. Natural products represent an attractive source of drug leads, as they have been selected through evolutionary processes to interact with biological targets.^1^ Indeed, most of the drugs approved for human use over the past decades are natural products or molecules derived from or inspired by natural products.^2^ For certain applications, such as immunosuppressants as well as antibacterial and anticancer agents, microorganisms have been the major contributors to our drug arsenal.^2^ The lion’s share among antimicrobial metabolites originates from strains belonging to the actinobacterial genus *Streptomyces*, accounting for more than two-thirds of antibiotics approved for human use.^3^

Over the course of several decades, millions of *Streptomyces* isolates have been screened for a variety of bioactive metabolites.^4^ Even though *Streptomyces* represent a highly exploited genus, recent genomic surveys project that only a small percentage of their specialized metabolites repertoire has been characterized.^5^ On average, *Streptomyces* strains harbor 20 to 30 biosynthetic gene clusters (BGCs) per genome,^5,6^ and each BGC can lead to a family of structurally related compounds. Under standard laboratory conditions, only some molecular families can be readily observed, either because of a lack of expression of the corresponding BGC,^7^ or inadequate detection methods. Some *Streptomyces* strains, however, display particularly generous metabolic profiles under standard laboratory conditions and have been recognized in the past as ″talented″ strains.^4^

In this paper, we describe the genome and metabolome of one particularly talented strain, emerged from a study aiming at examining the metabolic profiles of 20 *Streptomyces* strains under various culture conditions.^8,9^ The extracts prepared from *Streptomyces* strain ID71268 attracted our attention as they consistently presented a complex metabolic profile and inhibited the growth of the ESKAPE pathogen *Acinetobacter baumannii*. Herein, we report genomic and metabolomic analysis of this strain under a single experimental condition and identify the molecule responsible for the antibacterial activity against *A. baumannii* as an unusual tridecapeptide siderophore.

## Results and Discussion

### Genome Sequence of *Streptomyces* Strain ID71268

**Figure 1:**
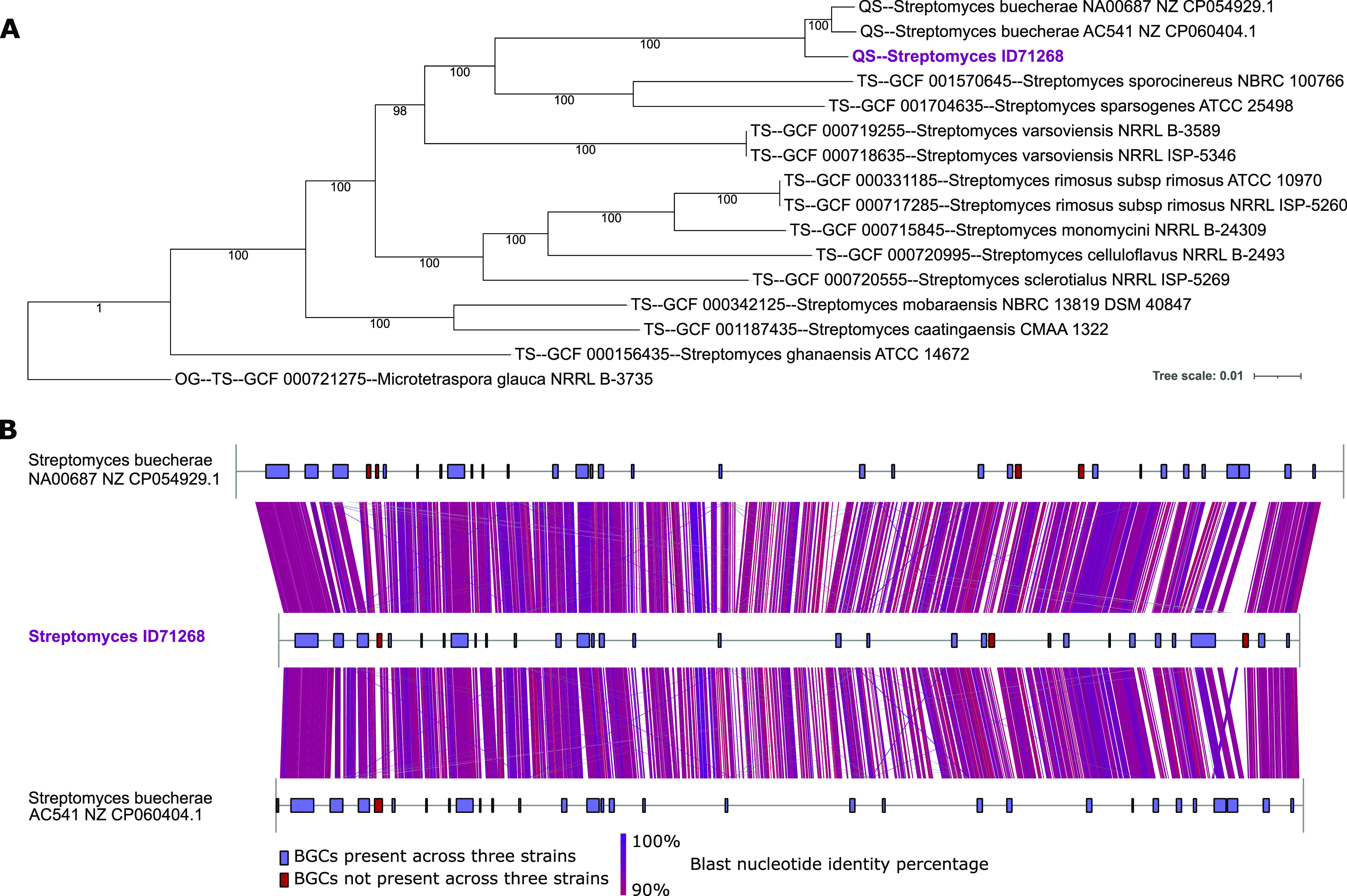
Panel A: multi-locus species tree generated with autoMLST, containing Streptomyces ID71268, two *S. buecherae* strains, and 12 closest strains selected with autoMLST. Panel B: full genome comparisons of Streptomyces ID71268 and two *S. buecherae* strains. Links indicate nucleotide identities >90%.

A draft genome of strain ID71268 was obtained as described in the Methods section. Its 8,379,354-bp genome available at NCBI BioProject ID PRJNA907813 presents the typical features of *Streptomyces* genomes (Table S1). The phylogenetic position of strain ID71268 was established using autoMLST,^10^ which indicated that it is closely related to *Streptomyces buecherae* strains AC541 and NA00687 (Figure 1A). The former is the type strain for this species, isolated in June 2014 from a female cave myotis bat (*Myotis velifer*) near Rattlesnake Springs in the Carlsbad Caverns National Park, California,^11,12^ while strain NA00687 was isolated from marine sediments in Hainan, China, and established to be a member of this species through average nucleotide identity.^12^ Remarkably, the genomes of the three strains show end-to-end similarity, with nucleotide identities over 90% for most of their genes (Figure 1B). According to our records, strain ID71268 was isolated in 1994 in the Lepetit laboratories from a soil sample collected in 1991 from an unspecified location in Colombia. Overall, these results suggest that strain ID71268 may also belong to the *S. buecherae* species and that members of this species can be found in diverse habitats.

When analyzed for the presence of BGCs through antiSMASH,^13^ strain ID71268 was found to contain 32 BGC-associated regions, for a total of 33 or 34 distinct BGCs, some of them highly related to known BGCs (Table 1). As predicted from the end-to-end genome similarity, just two and four small BGCs were absent in the genomes of strains AC541 and NA00687, respectively (Figure 1B), further strengthening the close relationships between these three isolates.

**Table 1:** Biosynthetic Gene Clusters Detected in the Genome of Streptomyces ID71268 by antiSMASH and Their Correspondence to Observed Metabolites

**region**	**type**	**class**	**start nt**	**end nt**	**most similar known cluster**	**similarity (%)**	**matched observed metabolites**
region 1	T1PKS	polyketide:modular type I	134,524	321,356	nigericin	100	nigericin
region 2	NRPS	NRP	451,427	529,430	dechlorocuracomycin	8	
region 3	NRPS	NRP	645,278	735,666	atratumycin	10	megalochelin
region 4	PKS-like, butyrolactone	polyketide	807,668	844,635	chalcomycin A	4	
region 5	redox-cofactor	polyketide	900,812	923,143	borrelidin	5	streptochlorin
region 6	butyrolactone		1,166,593	1,177,591			
region 7	siderophore		1,351,073	1,363,598			
region 8	T1PKS	polyketide	1,415,846	1,552,737	niphimycins C-E	41	azalomycins
region 9	RiPP-like		1,611,483	1,620,405			
region 10	RiPP-like		1,699,658	1,711,286			
region 11	siderophore	NRP	1,934,050	1,949,307	ficellomycin	3	
region 12	other, CDPS	NRP	2,274,950	2,317,086	BD-12	14	
region 13	T1PKS	polyketide	2,450,052	2,549,639	griseochelin	84	
region 14	terpene	terpene + polyketide:type III	2,568,290	2,588,015	merochlorin A/merochlorin B/deschloro-merochlorin A/deschloro-merochlorin B/isochloro-merochlorin B/dichloro-merochlorin B/merochlorin D/merochlorin C	7	
region 15	NRPS-like	NRP	2,631,275	2,671,249	echoside A/echoside B/echoside C/echoside D/echoside E	76	
region 16	terpene	terpene	2,907,252	2,927,188	xiamycin A	13	
region 17	terpene	terpene	3,608,209	3,629,393	geosmin	100	
region 18	arylpolyene	polyketide:iterative type I + polyketide:enediyne type I	4,572,658	4,613,875	kedarcidin	7	serpentene
region 19	CDPS, terpene		4,828,266	4,849,580			
region 20	NRPS	NRP:glycopeptide + saccharide:hybrid/tailoring	5,523,300	5,566,599	A40926	9	
region 21	ladderane	alkaloid	5,767,976	5,809,181	marinacarboline A/marinacarboline B/marinacarboline C/marinacarboline D	23	
region 22	NRPS	NRP	5,828,584	5,874,466	atratumycin	10	
region 23	terpene		6,315,096	6,335,429			
region 24	NRPS	NRP	6,442,687	6,485,070	diisonitrile antibiotic SF2768	66	diisonitrile antibiotic SF2768
region 25	ectoine	other	6,813,891	6,824,334	ectoine	100	ectoine
region 26	NRPS	NRP	6,984,391	7,028,025	paenibactin	83	DHB for megalochelin formation
region 27	NRPS, NAPAA	NRP:cyclic depsipeptide	7,195,934	7,237,870	stenothricin	13	
region 28	terpene	terpene	7,335,572	7,360,024	hopene	61	
region 29	PKS-like, trans-AT-PKS, T1PKS, terpene	polyketide	7,489,570	7,686,752	piericidin A1	100	29a: marinomycins, 29b: piericidin
region 30	NRPS, NRPS-like	other	7,913,092	7,956,862	spiroindimicin A/spiroindimicin B/spiroindimicin C/spiroindimicin D/indimicin A/indimicin B/indimicin C/indimicin D/indimicin E/lynamicin A/lynamicin D/lynamicin F/lynamicin G	9	
region 31	NRPS		8,043,210	8,091,630			
region 32	terpene		8,272,765	8,293,829			

### Metabolomic Analysis

**Figure 2:**
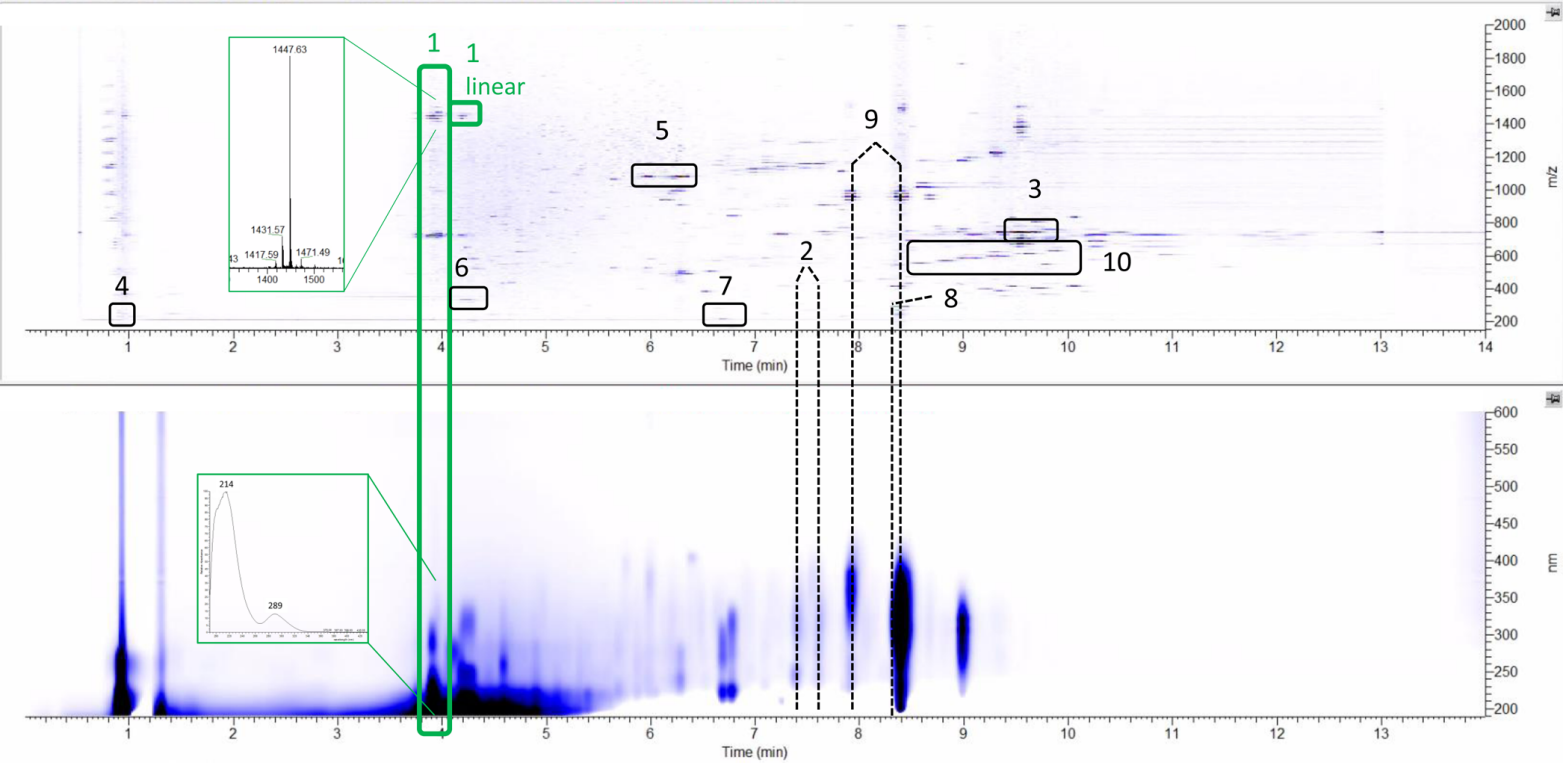
Typical metabolic profile of strain ID71268 as seen with a mass detector (top) and diode array detector (bottom). The numbered boxes and dashed lines indicate the metabolite families described in the text. The enlargements represent the MS (top) and UV (bottom) spectra of the observed compound.

A typical extract from strain ID71268, prepared as described in the Methods section, exhibited a complex metabolite profile, as exemplified in Figure 2. By analyzing data from DAD-equipped liquid chromatography coupled with an HR-MS instrument, we were able to identify several known metabolites by matching their fragmentation spectra with the ones obtained from authentic standards or by comparing their physicochemical behavior with data reported in the literature. Specifically, we identified: (Figures 2 and S1) three members of the piericidin family (box 2); the polyether nigericin (box 3); ectoine (box 4); three members of the azalomycin F family (i.e., 3a, 4a and 5a; box 5); and (Figure S2) the diisonitrile antibiotic SF2768 (box 6). The corresponding BGCs were easily identified as Regions 29b (piericidin), 1 (nigericin), 25 (ectoine), 8 (azalomycin F), and 24 (SF2768), each sharing a high percentage of similar genes with the cognate reference BGC (Table 1).

Furthermore, we identified a molecule (Figure 2, box 7) with a monoisotopic mass of 219.0319, an isotopic pattern indicative of a chlorine-containing compound and a UV spectrum with maxima at 272 and 287 nm (Figure S3A). These properties, along with the observed fragmentation pattern, are consistent with literature data reported for streptochlorin, a small, chlorinated compound with antiproliferative activity isolated from a marine *Streptomyces*.^14,15^ Since the streptochlorin BGC has not been reported, we searched the ID71268 genome for halogenase-encoding genes and found a single halogenase (ctg1_662) at the very right end of Region 5 (Table 1). The halogenase CDS is followed by a Na/H transporter, by a CDS related to a decarboxylase from the chondrochloren BGC,^16,17^ and by a CDS related to glucose/sorbose dehydrogenases. Two CDSs specifying proteins with no matches precede ctg1_662 (Figure S3B; Table S2). Overall, these data hint at the possibility that streptochlorin derives from tryptophan through decarboxylation or carboxyl migration, dehydrogenation, and chlorination.

An additional metabolite (box 8 in Figure 2) showed a UV spectrum with two maxima at 309 and 342 nm, a monoisotopic mass in positive ionization mode of 293.1536 [M + H]^+^, and a strong signal at *m/z* 291.1392 [M – H]^−^ in negative ionization mode (Figure S4A), suggesting the presence of an acid moiety. These data, as well as the fragmentation pattern, are in accordance with those reported for serpentene,^18^ a fully unsaturated carboxylic acid containing an *ortho*-substituted benzene ring isolated from *Streptomyces*. Serpentene is likely to be of polyketide origin^18^ and, being a highly symmetric molecule devoid of methyl branches, could originate from a T2PKS. Strain ID71268 contains just two such BGCs: Regions 4 and 18 (Table 1). Region 18 is a likely candidate for serpentene biosynthesis, since it encodes reductases and dehydratases, which are expected to be required to produce the polyene (Figure S4B, Table S3). Consistently, some portions of this BGC are highly related to the BGC for the formation of colabomycin,^19^ a highly unsaturated metabolite that also contains a C-6 aromatic ring. This finding suggests that serpentene and colabomycin may have a common biosynthetic origin. As a final note, Region 18 finds matches to enzymes involved in the synthesis of arylpolyenes, abundant metabolites functionally related to carotenoids but generally absent in actinomycetes.^20^

We also identified molecules (Figure 2, box 9) whose properties matched those of marinomycins A–D, dilactone polyene antitumor antibiotics discovered from a marine actinomycete:^21^ a characteristic UV spectrum with a maximum at 359 nm; monoisotopic masses of 997.5289, 997.5287, 997.5295, and 1011.6841; and consistent MS2 data (Figure S5A). Since the marinomycin BGC has not been reported yet, we tried to predict it bioinformatically among the antiSMASH-identified regions. Marinomycins consist of head-to-tail esters of two identical C_28_ polyketide chains, likely to be synthesized by a T1PKS. Additional features of these molecules include a single methyl branch in the polyketide chain at a β-carbon position, which is likely to originate from a β-branching mechanism,^22^ the total lack of fully reduced methylenes at the β-carbons, and a polyketide chain ending with a substituted benzoic acid unit. Table 1 lists only two possible T1PKS BGCs that do not yet have an assigned metabolite: Region 13 and Region 29a. The former is highly related to the BGC devoted to the synthesis of griseochelin, which is structurally unrelated to marinomycins. Consistently, the likely candidate—Region 29a—harbors a BGC encoding a 13-module PKS, mostly of the trans-AT type, two free-standing ATs, as well as the enzymes responsible for β-branching (Figure S5B, Table S4). The Region 29a PKS presents an unusual domain organization: there are six modules consisting of KS-ACP domains only, albeit marinomycins do not contain any β-keto groups, which suggests a trans-acting KR, with ctg1_6032 as a likely candidate; six modules consisting of KS-DH-KR-ACP domains, likely to install most of the double bonds in the marinomycin monomer; and a terminal module consisting of KS-AT-DH-TE domains (Figure S4B). The latter module, which contains the only cis-AT domain and a DH but not the expected KR domain, is highly related to AjuH, the terminal module in the biosynthesis of the myxobacterial metabolite ajudazol.^23^ Notably, ajudazol, although structurally unrelated to marinomycins, also ends with an aromatic C-6 ring. Thus, it is tempting to speculate that the aromatic ring is formed through a mechanism similar to that occurring in aromatic polyketide biosynthesis carried out by type II PKSs, with condensation between the C-2 carbon, flanked by two carbonyls, and the C-7 carbonyl, flanked by two double bonds, followed by DH-catalyzed C-2,C-6 dehydration. Overall, the order of the PKS modules in Region 29a would fit with a collinear arrangement with these biosynthetic steps (Figure S5B).

The antiSMASH analysis also reported additional regions with high similarity to known BGCs, such as those for the biosynthesis of geosmin, griseochelin, echosides, paenibactin, and hopene (Table 1). A targeted metabolomic analysis did not result in the identification of any of these molecules, indicating that under the cultivation conditions employed, griseochelin, echosides, hopene, and paenibactin-like compounds are not produced. Geosmin, if present, is likely to be lost during the extraction procedure.^24^

We also observed two families of putatively novel metabolites: a complex of lipophilic molecules (box 10 in Figure 2) with monoisotopic masses of 560.3791, 574.3940, 588.4092, 602.4250, and 616.4407 and poor UV absorption; and the metabolite (box 1 in Figure 2) described in the following sections. In both cases, no microbial metabolites matching the observed monoisotopic masses were found in databases.

**Figure 3:**
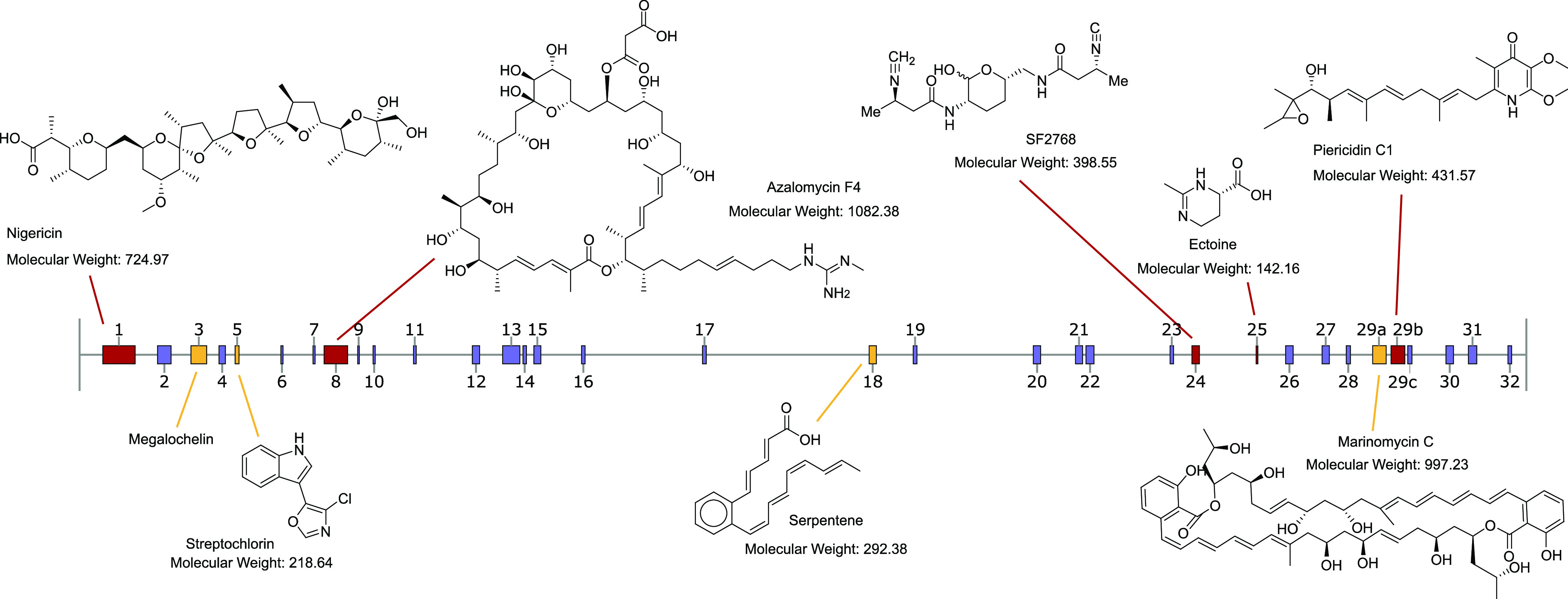
Schematic representation of the Streptomyces ID71268 genome displaying antiSMASH-predicted BGCs. Identified metabolites are illustrated above the genome (for simplicity, only one congener per family is shown), and the cognate BGCs present in the MiBIG database are in red color. The metabolites shown below the genome are linked to their cognate BGCs (in yellow) predicted in this work. The BGC number three represents the metabolite characterized and connected to the cognate BGC in this work.

Overall, we were able to identify 10 families of metabolites when strain ID71268 was analyzed in a single medium and at a single time point. At least five of the 10 metabolite families have a polyketide skeleton, indicating that strain ID71268 is a versatile producer of different metabolites, in particular polyketides. The identified metabolite families and the cognate BGCs are visualized in Figure 3.

### Metabolite Responsible for Activity against *A. baumannii*

When the same extract analyzed in Figure 2 was fractionated, only one fraction, highlighted in green, showed antibiotic activity against *A. baumannii*. Briefly, 3 mg of **1** was purified from 160 mL of culture as described in the Methods section. The molecular formula of **1** was established by high-resolution mass spectrometry as C_60_H_90_N_18_O_24_ (found *m/z* 1447.6444^+^ [M + H]^+^, calculated *m/z* 1447.6448 [M + H]^+^, 0.3 ppm mass difference; Figure S6A, upper spectrum). The UV maxima at 214 and 289 nm (Figure S6B) suggested the presence of a peptide backbone with aromatic moieties. Extensive one-dimensional (1D)- and two-dimensional (2D)-NMR experiments and HR-tandem MS analyses were then performed. ^1^H and ^13^C spectral data of **1** are reported in Table 2. The ^1^H NMR spectrum of **1** (Figure S7) showed the presence of signals in the aromatic and aliphatic regions, with an abundance of protons between 3.5 and 4.5 ppm, related to amino acidic α protons and hydroxy-methines or -methylenes. The presence of α protons was also supported by the bidimensional heteronuclear single quantum coherence (HSQC) experiment (Figure S12), where the above-mentioned signals presented cross peaks with carbons between 40 and 70 ppm. With the help of heteronuclear multiple bond correlation (HMBC) experiment (Figures S13 and S14A) and the overlap of homonuclear correlation spectroscopy (COSY) with total correlated spectroscopY (TOCSY) experiments (Figures S8, S9, and S11A), several proteinogenic amino acid spin systems were recognized and assigned as follows: three glycines, two threonines, two serines, one each leucine, valine, and glutamine. Moreover, the spin systems of two ornithines were observed, one in a cyclized-*N*-hydroxylated form (chOrn), the other as a *N*-formyl-*N*-hydroxy residue (fhOrn). Some HSQC cross-peaks in the 6.5–7.5 (proton) and 110–130 ppm (carbon) regions suggested the presence of aromatic moieties, but the signals did not match those from aromatic proteinogenic amino acids. In particular, two protons at 6.84 and 7.08 ppm on carbons at 119.6 and 116.4 ppm, respectively, showed in ^1^H monodimensional experiments a J-coupling of 8.5 Hz, suggesting they were in *ortho* position between each other on the same aromatic ring. Moreover, in the HMBC spectrum (Figure S14B), the proton at 7.08 ppm showed cross peaks with the nonprotonated carbons at 123.2 and 144.2 ppm, while the proton at 6.84 ppm showed cross peaks with the nonprotonated carbons at 120.2 and 147.1. These correlations are consistent with the presence of a 2,3 di-hydroxylated mono-substituted benzylic ring (DHB). When the HMBC correlations were analyzed from experiments optimized for two- and three-bond ^13^C-^1^H coupling constants (^2^J_CH_-couplings and ^3^J_CH_-couplings) (Figure S15AB), the data fitted substitution being at C-6. Thus, a 2,3 di-hydroxylated 6-substituted benzylic ring was present in **1**.

**Table 2:** NMR Data of Megalochelin

		**megalochelin**		
**unit**	**position**	**δH (multiplicity, J-coupling when visible (Hz))**	**δC**	**ROESY correlations**
2,3-DHB (dihydroxybenzoate)	C=O			
	1		120.2	
	2		144.2	
	3		147.1	
	4	7.08 (d, 8.5)	116.4	
	5	6.84 (d, 8.5)	119.6	
	6		123.2	
Gly_1	NH	7.86		
	C=O			
	αa	4.03	44.2	
	αb	3.99		
Gly_2	NH	8.59		4.03 (α-Gly_1)
	C=O		171.8	
	αa	4.12	42.9	
	αb	4.02		
Thr_3	NH	8.14		4.12 (α-Gly_2)
	C=O		173.3	
	α	4.15	60.7	
	β	4.02	66.5	
	γ	1.25	19.5	
Ser_4	NH	7.98		4.15 (α-Thr_3)
	C=O		171	
	α	4.35	57.2	
	βa	3.93	61	
	βb			
Leu_5	NH	7.49		4.35 (α-Ser_4)
	C=O			
	α	4.44	51.8	
	βa	1.63	40.6	
	βb			
	γ	1.66	24.4	
	δ	0.93	22.7	
	ε	0.88	19.9	
Thr_6	NH	7.53		4.44 (α-Leu_5)
	C=O		173.2	
	α	4.19	60.2	
	β	4.28	66.8	
	γ	1.27	18.6	
His_7	NH	8.35		4.19 (α-Thr_6)
	C=O		171.9	
	α	4.68	51.7	6.84 (5-DHB)
	βa	3.08	26	6.84 (5-DHB)
	βb	2.81		
	γ		weak	
	δ		weak	
	NH			
	ε	7.31	127.9	
Val_8	NH	7.95		4.68 (weak, α-Hys_7)
	C=O		171.8	
	α	4.12	59.4	
	β	2.07	30.3	
	γ	0.92	17.2	
	δ	0.93	18.5	
Gln_9	NH	8.54		4.12 (α-Val_8)
	C=O		172.6	
	α	4.34	53	
	βa	2.13	28.8	
	βb	1.95		
	γa	2.33	31.4	
	γb			
	C=O		176.2	
	NH2			
fhOrn_10	NH	8.36		4.34 (α-Gln_9)
	C=O			
	α	4.20	53.9	
	βa	1.82	23.4	
	βb	1.69		
	γa			
	γb			
	δa	3.55	49.6	
	δb			
	N–OH			
	formyl	7.95	157.7	3.55 (δ-f-OH-Orn_10)
Gly_11	NH	8.39		4.20 (α-f-OH-Orn_10)
	C=O			
	αa	3.95	42.5	
	αb	3.88		
Ser_12	NH	8.01		3.95 (α-Gly_11)
	C=O			
	α	4.43	59.3	
	βa	3.86	61.6	
	βb			
chOrn_13	NH	8.38		4.43 (α-Ser_12)
	C=O			
	α	4.48	50.3	
	βa	2.09	27	
	βb	1.95		
	γa	2.05	20.3	
	γb			
	δa	3.63	51.2	
	δb			
	N–OH			

An amino acid spin system involving an α methine (at 4.68 ppm on a carbon at 51.7 ppm) and β methylene protons (at 3.08 and 2.81 ppm on a carbon at 26 ppm) suggested the presence of a histidine moiety. However, a single additional aromatic proton at 7.31 ppm, bound to a carbon at 127.9 ppm, was detected in the HSQC spectrum, with the absence of clear HMBC cross peaks. In addition, rotating frame Overhauser effect spectroscopy (ROESY) (Figure S10) indicated that the α methine and β methylene protons mentioned above correlated to H-5 of DHB (Figure S11B,C), suggesting that the histidine residue is connected to the DHB moiety, in a C–C bond between His Cδ and DHB C-6.

ROESY experiments also established a partial amino acid sequence for **1** as: Gly-Gly-Thr-Ser-Leu-Thr-His as the *N*-terminal portion, followed by a weak ROESY cross peak connecting His to Val and then Gln-fhOrn-Gly-Ser-chOrn. Moreover, the presence of an amide signal in the Gly1 spin system suggested an amide bond with DHB.

**Figure 4:**
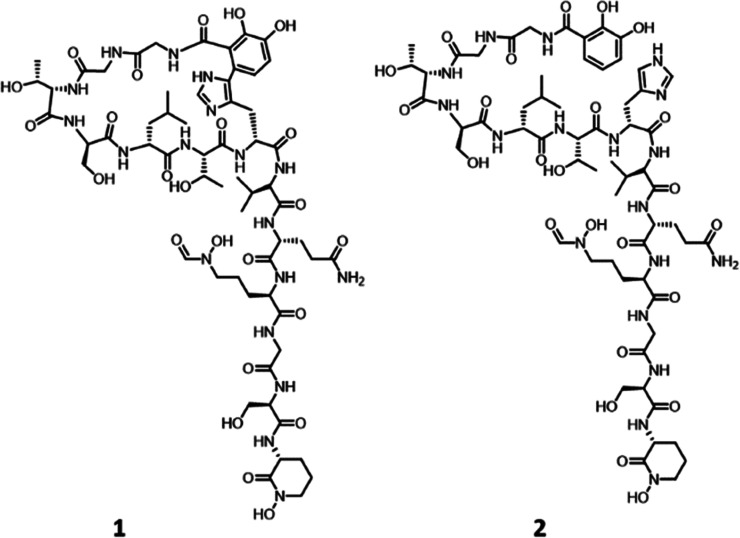
Structure of cyclized form of megalochelin (1) and its linear form (2).

Overall, these data are consistent with the structure shown in Figure 4, in which Gly1 is capped through an amide bond with DHB, which is then bound to His7 through a C–C link between DHB C-6 and His Cδ. High-resolution tandem mass spectral data (Figure S6C,D) fully confirmed the structure of **1**. In particular, the presence of fragments from b6 to b11 is fully consistent with the amino acid sequence from Val8 to chOrn13, while the absence of fragments among the amino acids from 1 to 7 strengthens the existence of a ring.

The same 160 mL culture also afforded about 1 mg of a partially purified, related metabolite **2** (corresponding to box “1 linear” in Figure 2), whose molecular formula was established by high-resolution mass spectrometry as C_60_H_92_N_18_O_24_ (found *m/z* 1449.6611^+^ [M + H] ^+^, calculated *m*/*z* 1449.6605 [M + H] ^+^, 0.4 ppm mass difference; Figure S6A, lower spectrum), thus differing from **1** by exactly one unsaturation. While complete NMR analysis of **2** was not possible, critical differences were observed in the aromatic region of the HSQC/HMBC overlay, where the entire spin system of His7 appears (green boxes in Figure S14C), together with the presence of a third proton in the DHB spin system (orange boxes in Figure S14C). These data are consistent with **2** being the linear form of **1**, thus lacking the C–C bond connecting DHB to His7. Consistently, the fragmentation spectrum of **1** and **2** were superimposable in the Val8–chOrn13 portion. However, high-resolution tandem mass spectral data of **2** (Figures S6C and S6E) revealed all of the expected fragments between Gly1 and His7, confirming that **2** is the linear form of **1**, thus corroborating the amino acid sequence from Gly1 to His7 established for **1** on the basis of NMR data alone.

The stereochemistry of the amino acid building blocks in **1** was established using Marfey’s method.^25^ Only threonine and valine residues were found to be in *L*-configuration, while all of the other amino acids (Ser, Leu, Gln, and Orn) were in the *D*-configuration (Figure S16). Since no commercial standards were available to establish the configuration of the DHB-His moiety, we resorted to processing **2**. The results indicated that His is also in the *D*-configuration (Figure S16). Assuming that the cross-link between DHB and His does not affect the configuration at the His α-carbon, the structure of **1** could be fully resolved at all its stereocenters, as shown in Figure 4.

The presence of fhOrn and chOrn residues suggested that **1** binds iron. Indeed, liquid chromatography–mass spectrometry (LC-MS) analyses clearly indicated that **1** readily forms a complex with Fe^3+^ (Figure S6F) and Al^3+^ but not with Zn^2+^. Further analyses (Figure S17) indicated that the tail portion of **1** still retains the ability to bind Fe^3+^. Thus, the function(s) and role of the ring in **1** await further studies.

The structure of **1** is unique for three main reasons. First, to our knowledge, there are no precedents of iron chelators with a ring-and-tail structure. Second, the ring is formed by a C–C bond connecting the first and the seventh amino acid in a very particular confirmation—a biosynthetic task likely requiring a highly specific enzyme and, consequently, a valid biological reason. Third, its size: the Natural Products Atlas^26^ lists 296 entries containing either “chelin,” “ferrin,” or “bactin.” The largest molecule among those entries is Azotobactin delta,^27^ a 1393-Da peptidic siderophore of NRPS origin^28^ from *Azotobacter vinelandii*, rendering **1** the largest siderophore ever described. Hence, we named the compound megalochelin.

### Megalochelin Antibacterial Activity is Iron-Dependent

**Figure 5:**
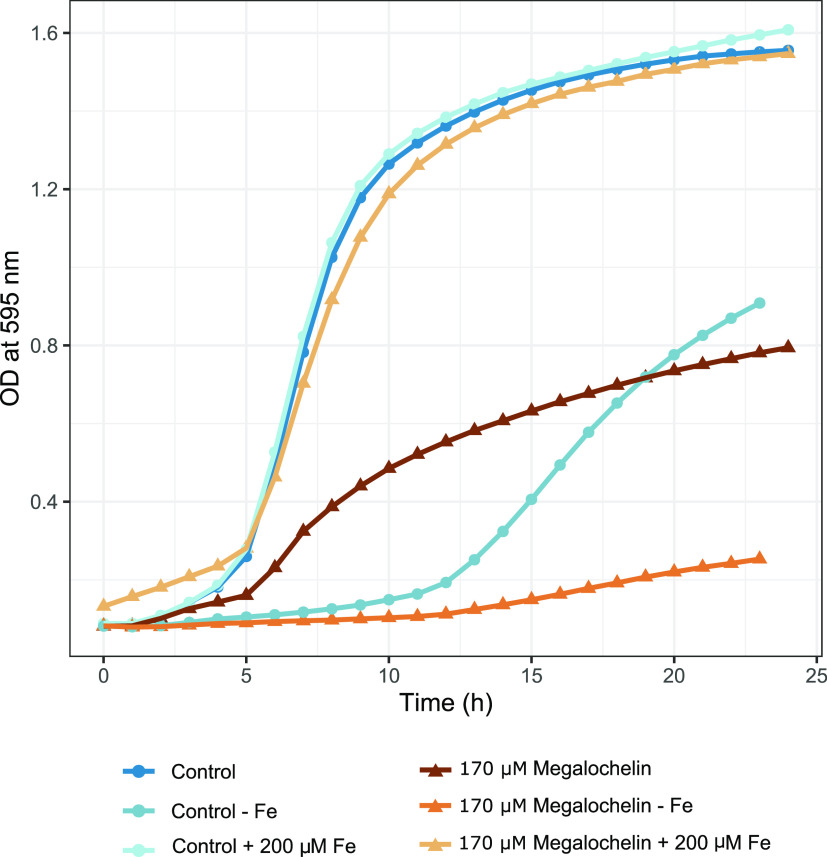
Effect of megalochelin on growth of A. baumannii. The figure shows the effect of 172 μM megalochelin in Mueller Hinton broth with and without 200 μM FeCl3 added, and in iron-limited medium without megalochelin or with 172 μM megalochelin added.

In the complex medium Müller–Hinton, megalochelin significantly affected the growth rate of *A. baumannii*. For example, in the presence of 172 μM **1**, *A. baumannii* growth was about 40% of control at most time points (Figure 5). Adding 200 μM FeCl_3_ to this medium completely abolished the inhibitory effect (Figure 5). In modified Davis minimal medium (no iron added), the strain could grow after an extensive lag, reaching an OD_600_ of 0.9 after 23 h. In this medium, growth of *A. baumannii* in the presence of 172 μM megalochelin was 60–70% lower relative to the control (Figure 5). These data indicate that megalochelin interferes with growth in an iron-dependent manner, with no effect seen in the presence of excess iron and increased inhibitory activity in iron-depleted medium. A clear growth inhibitory effect in Müller–Hinton broth could be seen up to 22 μM megalochelin (Figure S18).

We also observed growth inhibition of the Gram-positive bacteria *Micrococcus luteus* and *Staphylococcus aureus* and, to a very limited extent, *Pseudomonas aeruginosa* (Figure S18). No significant effect was seen with other human pathogens (*Escherichia coli*, *Enterococcus faecium*, *Klebsiella pneumoniae*, or *Streptococcus pneumoniae*; Figure S18).

It is important to note that while siderophores are common in actinomycete-derived extracts (e.g., refs^9,24^^9, 24^), inhibition of *A. baumannii* growth was observed infrequently (ref (9), M.I, unpublished data), suggesting that not all siderophores interfere with the growth of this species. In fact, several siderophores have been shown to enhance the growth of *A. baumannii,*(29,30) and this species is known to take up and thrive on xenosiderophores,^31^ i.e., siderophores produced by other bacterial or fungal species. In addition, *A. baumannii* has evolved to cope with nutritional immunity by developing multiple mechanisms for iron sequestration;^32,33^ for example, human pathogenic strains are capable of producing several types of siderophores such as fimsbactin, baumanoferrin, and acinetobactin.^34^ Finally, some siderophores that separately promote the growth of *A. baumannii* can have an opposite effect if combined.^30^ Thus, the interplay between siderophores and microorganisms is a complex trait, and further studies will be necessary to understand megalochelin’s effects on *A. baumannii* growth.

### Analysis of the Megalochelin Biosynthetic Gene Cluster

Region 3 from strain ID71268 genome is an obvious candidate for specifying the biosynthesis of megalochelin, since it encodes a 13-module NRPS within a 65-kbp region designated as the *mcl* (*m*egalo*c*he*l*in) BGC. The genes upstream of the NRPS genes *mclGIJ* include (Table S5) a pair of divergently transcribed genes (*mclA* and *mclB*) annotated as lysine/ornithine monooxygenase and methionyl-tRNA formyltransferase, respectively, likely to be involved in *N*-hydroxylation of Orn10 and Orn13 and in formylation of Orn10, respectively; a pair of iron chelate uptake ABC transport genes (*mclC* and *mclD*); a MbtH-like protein (*mclE*); and *mclF*, annotated as a multicopper oxidase type 2. The latter is highly related to genes from the *Salinispora arenicola* rifamycin^35^ and kanglemycin BGCs.^36,37^ To our knowledge, the function of these oxidases in rifamycin or kanglemycin biosynthesis has not been established. Nonetheless, multicopper oxidases belong to the laccase family, enzymes involved in oxidizing phenolic compounds and making cross-links.^38^ Thus, it is tempting to speculate that MclF might be involved in cross-linking the catechol and histidine moieties. Downstream of the NRPS genes are three genes annotated as ABC transporters *(mclJLM)* and *mclK*, which is annotated as vibriobactin utilization protein ViuB. So, *mclCDJLKM* are likely involved in the transport of megalochelin (Table S5).

Figure S19 showcases the comparison between the *mcl* BGC, 10 most similar BGCs identified by antiSMASH analysis, and one BGC identified in *S. buecherae* strain AC541. In addition to those from the two *S. buecherae* strains, the BGC was present and highly conserved in a clade of seven genomes, including *S. griseus* NRBC 13350. Interestingly, all analyzed regions share four genes upstream to and four genes downstream of the NRPS genes, suggesting that the BGC core region encompasses at least 11 genes. Of note, all BGCs encode a multicopper oxidase protein, and none of them encode any regulator, suggesting the BGCs might be constitutively expressed.

**Figure 6:**
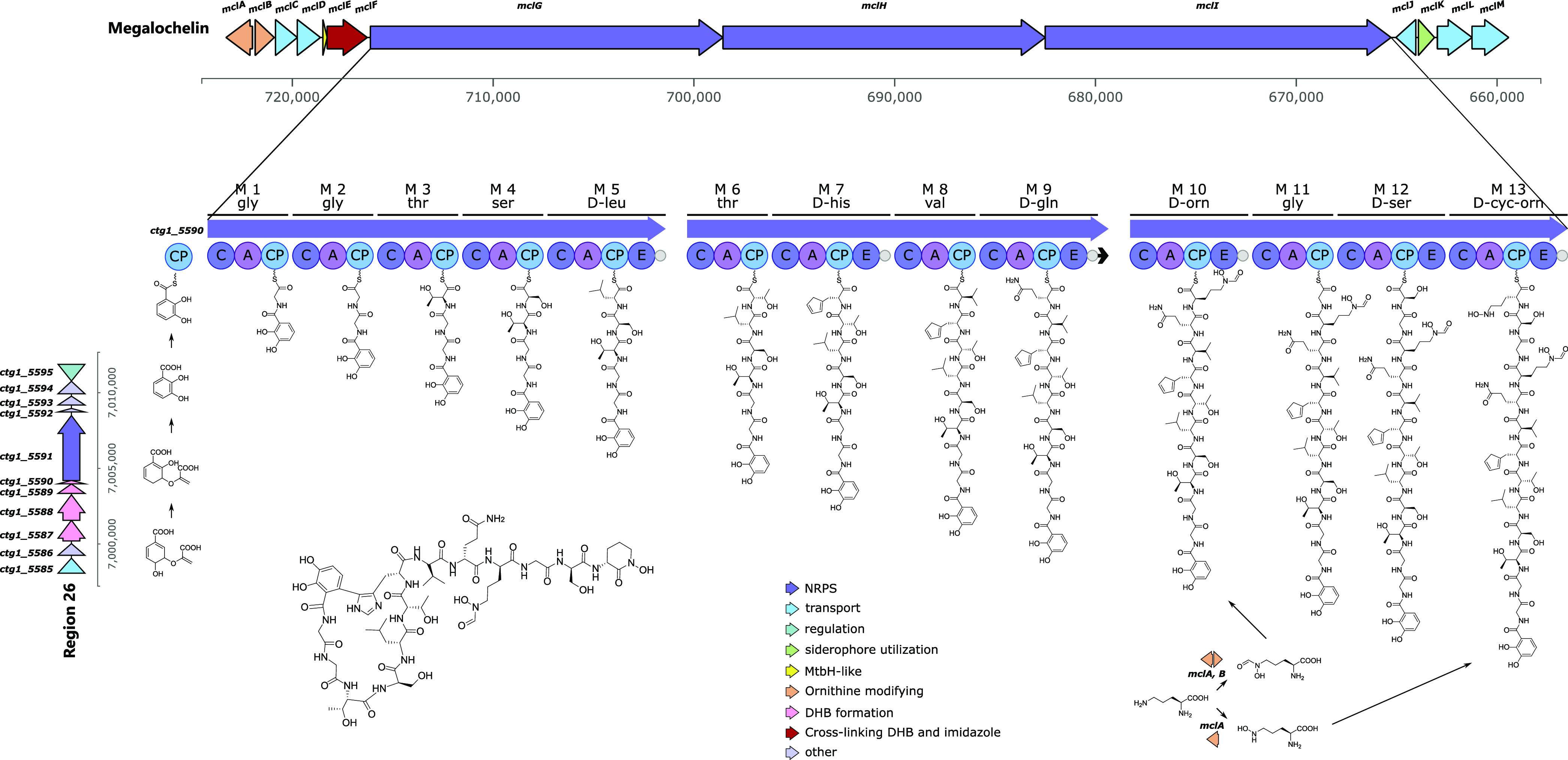
Megalochelin formation. Top: BGC encoding the peptide chain of megalochelin. Peptide chain elongation is shown from left to right on NRPS modules. Left: BGC encoding the DHB starter unit. Genes are color-coded by function. A full list of genes and functions is found in Tables S5 and S6.

The megalochelin NRPS is split over three large polypeptides containing five, four, and four modules, respectively (Figure 6). All polypeptides start with a C-domain, and no module contains a TE domain, as also found in the chOrn-ending siderophore qinichelin.^25^ The amino acid specificities could be bioinformatically predicted for only 8 out of 13 A-domains. For the remaining A-domains, the specificities could, in some cases, be inferred by comparison with established A-domains (data not shown). Accordingly, the first four modules in MclG are likely to incorporate Gly, Gly, Thr, and Ser, respectively, suggesting that this NRPS is involved in starting the polypeptide chain. Consistently, the first C-domain in MclG is highly related to C-domains using a DHB as a starter unit (Figure S20). The fifth module in MclG contains an E-domain, and its A-domain is likely to incorporate a hydrophobic amino acid. The first and third modules of MclH, which lack E-domains, are predicted to incorporate Thr and Val. While no amino predictions were possible for the second and fourth modules of MclH, each of them contains an E-domain. This is consistent with MclH extending the MclG-formed pentapeptide with Thr-His-Val-Gln, with His and Gln epimerized to the *D*-forms. Finally, the predicted specificities of the second and third A-domains of MclI are Gly and Ser, and all modules in MclI except the second carry an E-domain. This suggests that MclI is involved in completing the tridecapeptide installing D-fhOrn, Gly, D-Ser, and D-hOrn, followed by chain release through amide bond formation. Overall, the A-domain-predicted specificities and the presence of E-domain appear to be collinear with the megalochelin structure with one exception: both Ser residues were experimentally found to be in the *D*-configuration (Figure S16), but the predicted Ser-specific module 4 does not contain an E-domain. Further studies will be necessary to understand the mechanism through which *D*-Ser4 is installed.

The hypothesized biosynthetic pathway for megalochelin is depicted in Figure 6. Cyclization of the DHB and His moieties may occur while the peptide is still bound to the NRPS or after peptide release, and the observed small amount of linear form can be due to incomplete cross-linking at either stage. The hypothetical pathway depicted in Figure 6 requires the supply of an activated form of DHB, to be condensed with glycine by the first NRPS module. While the megalochelin BGC harbors no such genes, a DHB-synthesizing cassette is found in Region 26 (Table S6), which is expected to specify the formation of an enterobactin-type monomer (DHB-Ser). A precedent for ″in-trans″ supply of DHB has been documented for qinichelin where the DHB-synthesizing cassette is also associated with an enterobactin-like BGC.^25^

## Conclusions

The work presented here provides unexpected findings about *Streptomyces* biology and siderophores. Strain ID71268 is a prolific producer of different metabolites, in particular polyketides. The type strain of this species was isolated from cave bats and was reported to exhibit potent antifungal activity, which might protect bats from the fungal pathogen *Pseudogymnoascus destructans*.^11^ While we are not aware of any metabolic studies on the *S. buecherae* strains, at least three of the metabolites produced by ID71268 possess antifungal activity: azalomycin F,^39^ streptochlorin,^14,40^ and SF2768.^41^ It would be interesting to establish whether other *S. buecherae* isolates produce a rich variety of different metabolite families. Our work has also identified tentative BGCs for serpentene, streptochlorin, and marinomycins, highlighting the power of combining metabolite annotation with whole genome sequencing in establishing likely BGCs for ″orphan″ metabolites.

Another unexpected finding is the unique structure of megalochelin. To our knowledge, this molecule represents the first example of a ″ring-and-tail″ siderophore, in which the tail is involved in iron binding, while the function of the 8-membered ring is not currently known. Ring-and-tail systems are a feature of different other families of natural products, where the rigidity offered by the macro ring system may allow binding to particular receptors, with the tail exerting a different role. Rigidity of the structure could also play a role in bioactivity: the DHB cap on Gly1 and the imidazole moiety of His7 closely resemble the termini of acinetobactin, which can be turned into a growth inhibitor by increasing the rigidity of the structure.^42^

## Methods

### General Experimental Procedures

^1^H and ^13^C 1D and 2D NMR spectra (COSY, TOCSY, NOESY, ROESY, HSQC, HMBC) were measured in CD_3_CN:D_2_O 8:2 or in DMSO_d6_ with or without drops of H_2_O at 25 °C using a Bruker Avance II 300 MHz spectrometer.

LC-MS/MS analyses were performed on a Dionex UltiMate 3000 HPLC system coupled with an LCQ Fleet (Thermo Scientific) mass spectrometer equipped with an electrospray interface (ESI) and a tridimensional ion trap. The column was an Atlantis T3 C-18 5 μm × 4.6 mm × 50 mm maintained at 40 °C at a flow rate of 0.8 mL/min. Phase A was 0.05% trifluoroacetic acid (TFA), and phase B was 100% acetonitrile. The elution was executed with a 14 min multistep program that consisted of 10, 10, 95, 95, 10, and 10% phase B at 0, 1, 7, 12, 12.5, and 14 min, respectively. UV–vis signals (190–600 nm) were acquired using a diode array detector. The *m**/**z* range was 110–2000, and the ESI conditions were as follows: spray voltage of 3500 V, capillary temperature of 275 °C, sheath gas flow rate at 35 units, and auxiliary gas flow rate at 15 units.

LC-HRMS/MS analyses were performed using an UHPLC system (Vanquish, Thermo Scientific) with a YMC-Triart ODS column (3.0 × 100 mm, S-1.9 μm, 12 nm) coupled to an Orbitrap Exploris 120 high-resolution mass spectrometer (Thermo Scientific, San Jose, CA). The mobile phase consisted of 0.1% formic acid in H_2_O (A), LCMS grade acetonitrile (B), and LCMS grade isopropyl alcohol (C). The gradient program was as follows: 0–1 min, 10% B and 0% C, 1–12.5 min, 95% B and 0% C, 12.5–13 min, 35% B and 60% C, 13–17 min, 35% B and 60% C, 17–18 min, 95% B and 0% C, 18–18.5 min, 10% B and 0% C, 18.5–23 min, 10% B and 0% C. The flow rate was 0.8 mL min^–1^, and we used an 8-μL injection volume at 40 °C. After chromatographic separation, the flow was split using a zero dead volume T-splitter (Valco), with 75% flow going to the Diode Array detector—acquiring data between 190 and 600 nm at a bandwidth of 1 nm—and 25% to the mass spectrometer. The latter was equipped with a heated electrospray ionization source (HESI, Washington, DC) operating in positive and negative ionization modes. Instrument calibration was carried out every analytical session with a direct infusion of a Pierce FlexMix calibration solution (Thermo Scientific, San Jose, CA). Ion transfer tube temperature and vaporizer temperature were set at 290 and 280 °C, respectively; the HESI spray voltage was 3.5 and 3.0 kV in positive and negative modes, respectively; sheath and auxiliary gas were set at 45 and 12 arbitrary units, respectively. Each experiment was acquired using the RunStart EASY-IC method for internal mass calibration. Data-dependent acquisition was performed using the following parameters: MS1 resolution was set at 60,000 with a normalized automatic gain control (AGC) target at 50%, a maximum inject time set to auto, and a scan range from 150 to 2000 *m*/*z*. For MS2, resolution was set at 30000 with a normalized AGC target of 100%, with a maximum inject time of 70 ms. The top 4 abundant precursors within an isolation window of 1.5 *m*/*z* were considered for MS/MS analysis. Dynamic exclusion was set at 3.5 s. Mass tolerance of ±5 ppm was allowed, and the precursor intensity threshold was set at 1 × 10^5^. For precursor fragmentation in higher-energy C-trap dissociation mode, a stepped collision energy mode was chosen, with normalized collision energies of 15–30–80%. After four data-dependent scans, a second MS1 experiment was acquired using the following parameters: resolution was set at 60,000 with a normalized AGC target at 50%, a maximum inject time set to auto, and a scan range from 180 to 2000 *m*/*z*.

### Metabolite Databases

For metabolite identification, we used an in-house proprietary compound library named ABL together with online resources (Natural Products Atlas, PubChem, ChemSpider).

### Megalochelin Purification

For the production of **1**, 1.5 mL of the −80 °C stock culture was inoculated into 2 × 15 mL of AF medium (dextrose monohydrate 20 g/L, yeast extract 2 g/L, soybean meal 8 g/L, NaCl 1 g/L, and CaCO_3_ 3 g/L, pH 7.3, prior to autoclaving) in one 50 mL baffled flask and grown for 3 days at 30 °C at 200 rpm. The AF cultures were transferred into one 0.5 L baffled flask containing 160 mL of M8 medium (dextrose monohydrate 10 g/L, yeast extract 2 g/L, meat extract 4 g/L, soluble starch 20 g/L, casein 4 g/L, CaCO_3_ 3 g/L, pH 7.2, prior to autoclaving) containing 5% (v/v) HP20 resin. The culture was harvested at 72 h and centrifuged, and the pellet (containing mycelium plus resin) was extracted with 64 mL of methanol by leaving it on a tilting shaker at room temperature for 1 h. After centrifugation for 10 min at 3000 rpm, the supernatant was transferred into a 500 mL glass bulb and dried under reduced pressure using a rotavapor. The residue, which contained about 5 mg of **1** and 2 mg of **2**, was resuspended in 2 mL of 30% dimethylformamide and resolved using a CombiFlash RF (Teledyne ISCO) medium-pressure chromatography system on a 12 g reversed-phase Biotage SNAP Ultra C18 25 μm cartridge. Flow was 12 mL/min, phase A was formic acid 0.05%, and phase B was acetonitrile. The column was previously conditioned with 5% phase B for 3 min, followed by a 33 min gradient to 70% phase B and 5 min gradient to 90% phase B. Fractions of 15 mL were collected and analyzed by LC-MS. Fractions with similar purity were pooled and dried under vacuum, yielding about 3 mg **of 1** and less than 2 mg of a mixture of **1** and **2** in a 3:7 ratio. These samples were used for NMR characterization, chemical analysis, and biological assays.

### Stereochemistry Determination

About 1 mg of **1** and 0.5 mg of **2** were treated as described,^25^ together with the following amino acid standards: *L*-Thr, *D*-Thr, *L*-*allo*-Thr, *D*-*allo*-Thr, *L*-Val, *D*-Val, *L*-Leu, *D*-Leu, *L*-Gln, *D*-Gln, *L*-Ser, *D*-Ser, *L*-Orn, *D*-Orn, *L*-His, and *D*-His and then analyzed by LC-MS using a HiQ sil C18 HS column (4.6 × 250 mm, Kya Tech Corporation, Japan). Phase A was 0.05% trifluoroacetic acid, and phase B was acetonitrile. The samples were run using a gradient from 10 to 90% B in 45 min at a flow rate of 0.8 mL/min.

### Metal-Binding Experiments

Semi-purified **1** in 50% MeCN was mixed in 1:1 ratio with sterile filtered saturated solutions of FeCl_3_, AlCl_3_, or ZnCl_2_. The formation of the expected metal adduct was observed by LC-MS; MS/MS data provide an indication of the metal-binding fragments.

### Genome Sequencing and Bioinformatic Analyses

Genomic DNA of Streptomyces strain ID71268 was prepared from a 5 mL overnight culture in AF medium. After centrifugation at 3000 rpm for 10 min, the pellet was washed with 10 mL of 10.3% sucrose to remove media traces and resuspended in 5 mL SET buffer. The cell wall was slowly digested by adding 50 mg of lysozyme powder three times every 3 h, incubating tubes at 37 °C overnight. The next day, we added 75 μg of RNAse A and, after 2 h at 37 °C, 3 mg of proteinase K, incubating for one further hour. Then, SDS was added to a final concentration of 1%, followed by 1 h at 55 °C. After adding 2 mL of 5M NaCl, the mixture was treated with 4 mL of phenol-chloroform-isoamylalcohol at pH 8 by inverting and gentle mixing, followed by centrifugation at 4000 rpm for 10 min. The aqueous phase was transferred to a new Falcon tube, and phenol was removed by adding 4 mL of chloroform-isoamylalcohol, followed by gentle mixing by inversion and centrifugation as before. The aqueous phase was transferred to a new 15 mL Falcon tube, and DNA was precipitated using ice-cold isopropanol. The DNA was spooled with a glass rod and washed with 70% EtOH. The material was dried on a thermorack, resuspended in 10 mM TRIS–HCl pH 8.5, and stored at +4 °C until sequencing.

Full genome sequencing with both Illumina and Oxford Nanopore Technology (ONT) was carried out at MicrobesNG, UK. The final genome sequence was obtained by first assembling the ONT long reads and then improving the accuracy by mapping the short Illumina reads on top, using the following software in the following order: guppy v3.03, flye v2.5, racon v1.4.7, medaka v0.7.1, bowtie2, and pilon v1.23. This resulted in one contig in 10 segments, nine of which were omitted as less than 400-nt long. The remaining 8,379,354-nt contig was manually edited in the *mcl* BGC (three frameshifts were observed) and deposited at NCBI database under accession number PRJNA907813.

The BGCs in the genome assembly were predicted using antiSMASH.^43^ The phylogenetic position of *Streptomyces* ID71268 was established using autoMLST.^10^ The full genome comparisons between *Streptomyces* ID17268 and *S. buecherae* strains AC541 and NA00687 were plotted with Easyfig.^44^ The megalochelin BGC comparison figures were created using clinker.^45^ The closest C-domains to the first C-domain of megalochelin were detected using NaPDoS.^46^

### Antibacterial Activity

The antibacterial activity of compound **1** was determined against strains from the NAICONS collection of bacterial pathogens as follows: 90 μL of 1 × 10^5^ CFU/mL bacterial suspension was dispensed into each well of a 96-well plate containing 10 μL of serial 1:2 dilutions of **1** (from a 10 mg/mL stock solution in 40% DMSO). Media were cation-adjusted Müller–Hinton or Davis–Mignoli (ammonium sulfate 1 g/L, dextrose 1 g/L, K_2_HPO_4_ 7 g/L, KH_2_PO_4_ 2 g/L, MgSO_4_ x 7 H_2_O 0.1 g/L, sodium citrate dihydrate 0.5 g/L, l-arginine 0.02 g/L, l-tryptophane 0.02 g/L). Plates were incubated in a Synergy 2 (Biotek) microplate reader with readings at 595 nm registered every hour.

## Supporting Information Available

Metabolite identification, BCG and metabolite matching, structural characterization of megalochelin and its biological activity, and tables of genes in the BGCs matched with corresponding metabolites (PDF)
