# Supplementary material for: Megalochelin, a Tridecapeptide Siderophore from a Talented Streptomycete
Source: ACS Chem Biol. 2023 Mar 15;18(4):861–74. doi: 10.1021/acschembio.2c00958 (PMC10127220; doi:10.1021/acschembio.2c00958)
Supplement: Supplementary file 1 — cb2c00958_si_001.pdf [file cb2c00958_si_001.pdf]

Supporting Information

Megalochelin, a tridecapeptide siderophore from a talented streptomycete

Kristiina Vind*[,†,‡], Cristina Brunati[†], Matteo Simone[†], Margherita Sosio[†], Stefano Donadio[†] and Marianna Iorio*[,†]

[†] NAICONS Srl, 20139 Milan, Italy

[‡] Host-Microbe Interactomics Group, Wageningen University, 6708 WD Wageningen, The Netherlands

* Corresponding authors

Kristiina Vind, email: kristiina.vind@wur.nl

Marianna Iorio, email: miorio@naicons.com

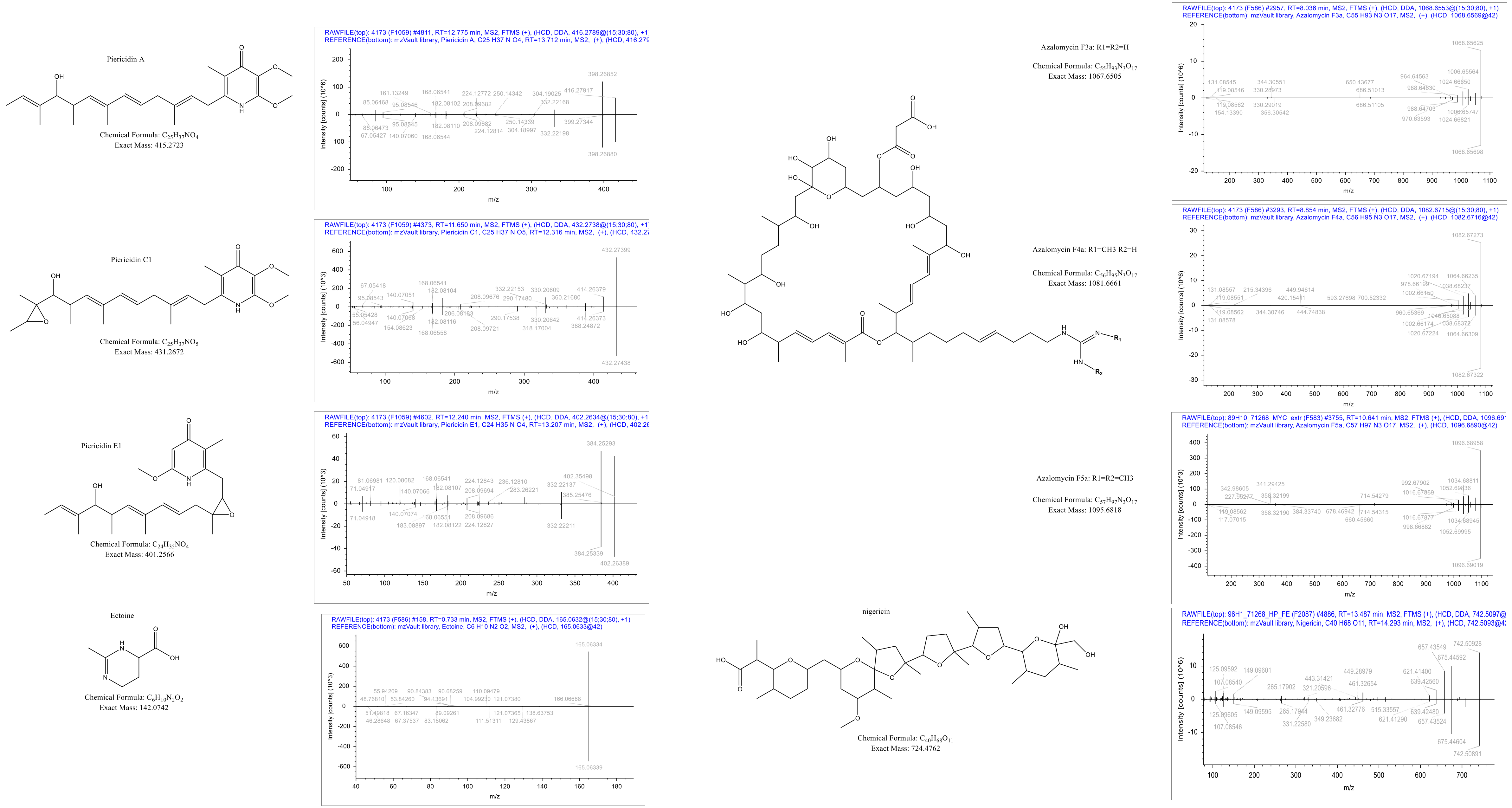

Figure S1. Selection of dereplicated metabolites identified mainly based on MS/MS fragmentation patterns. Left: name, structure, chemical formula and exact mass, right: their relative butterfly plots matching the authentic standard molecule. For all the reported structures stereochemistry is assumed to be as reported in Natural Product Atlas (https://www.npatlas.org/).

A

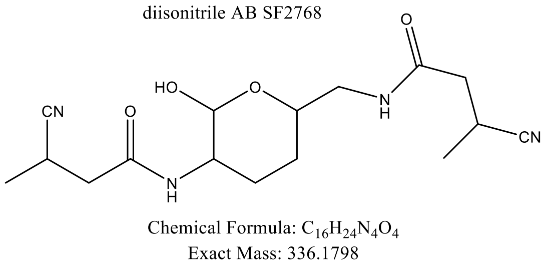

B

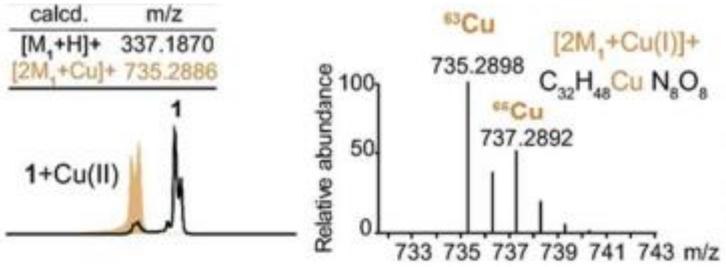

From: Wang L., et al. Diisonitrile Natural Product SF2768 Functions As a Chalkophore That Mediates Copper Acquisition in *Streptomyces thioluteus*. ACS Chem Biol. 2017 Dec 15;12(12):3067-3075. doi: 10.1021/acschembio.7b00897. Epub 2017 Nov 22. PMID: 29131568.

C

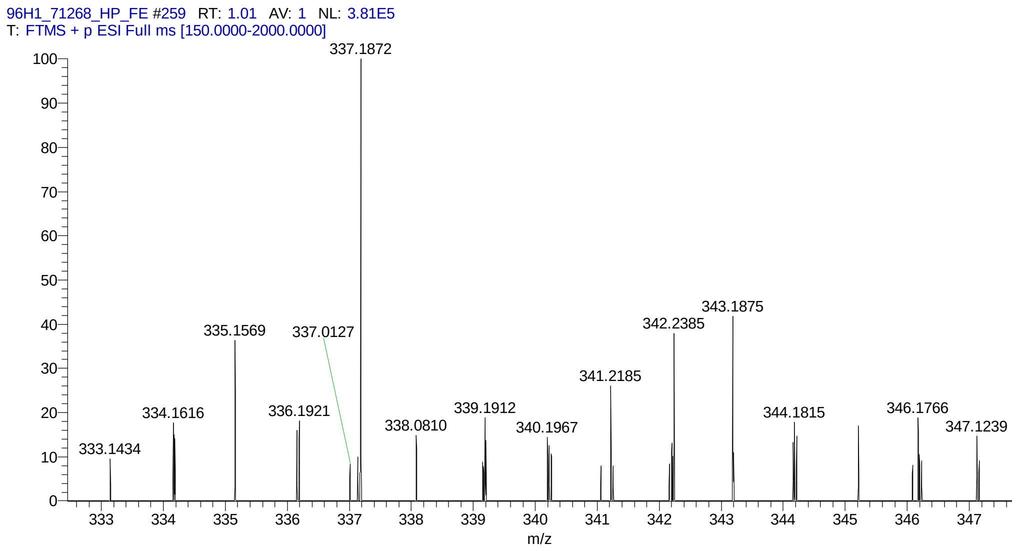

D

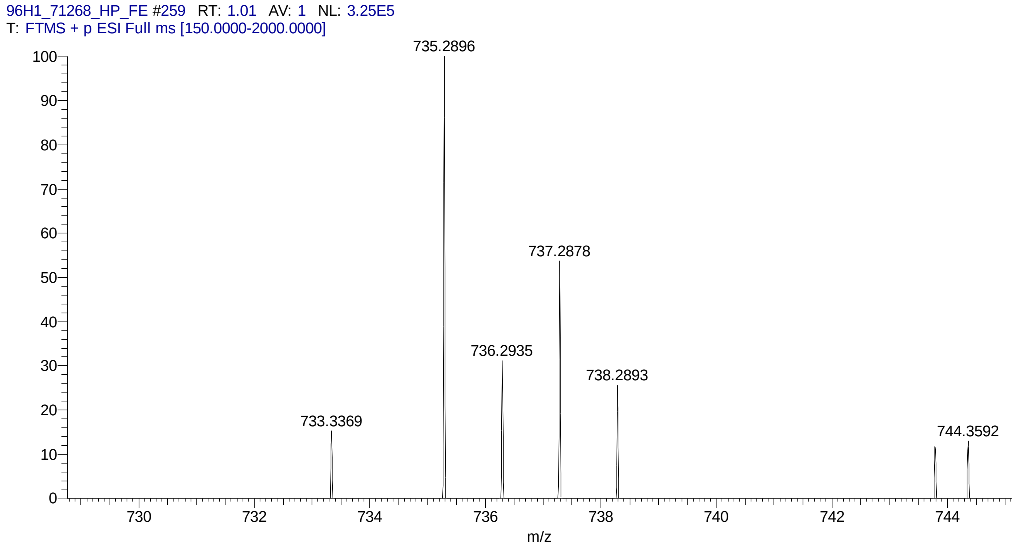

Figure S2. DiisonitrileAB SF2768: A) structure; B) revisited figure 5 from Wang et al. (2017) showing the isotopic pattern of diisonitrileAB SF2768 complexed with Cu(I)+, C-D) HR full masses in positive ionization mode showing *m/z* 337.1872 [M+H]+ (C)and *m/z* 735.2896 [2M+Cu(I)]+ (D).

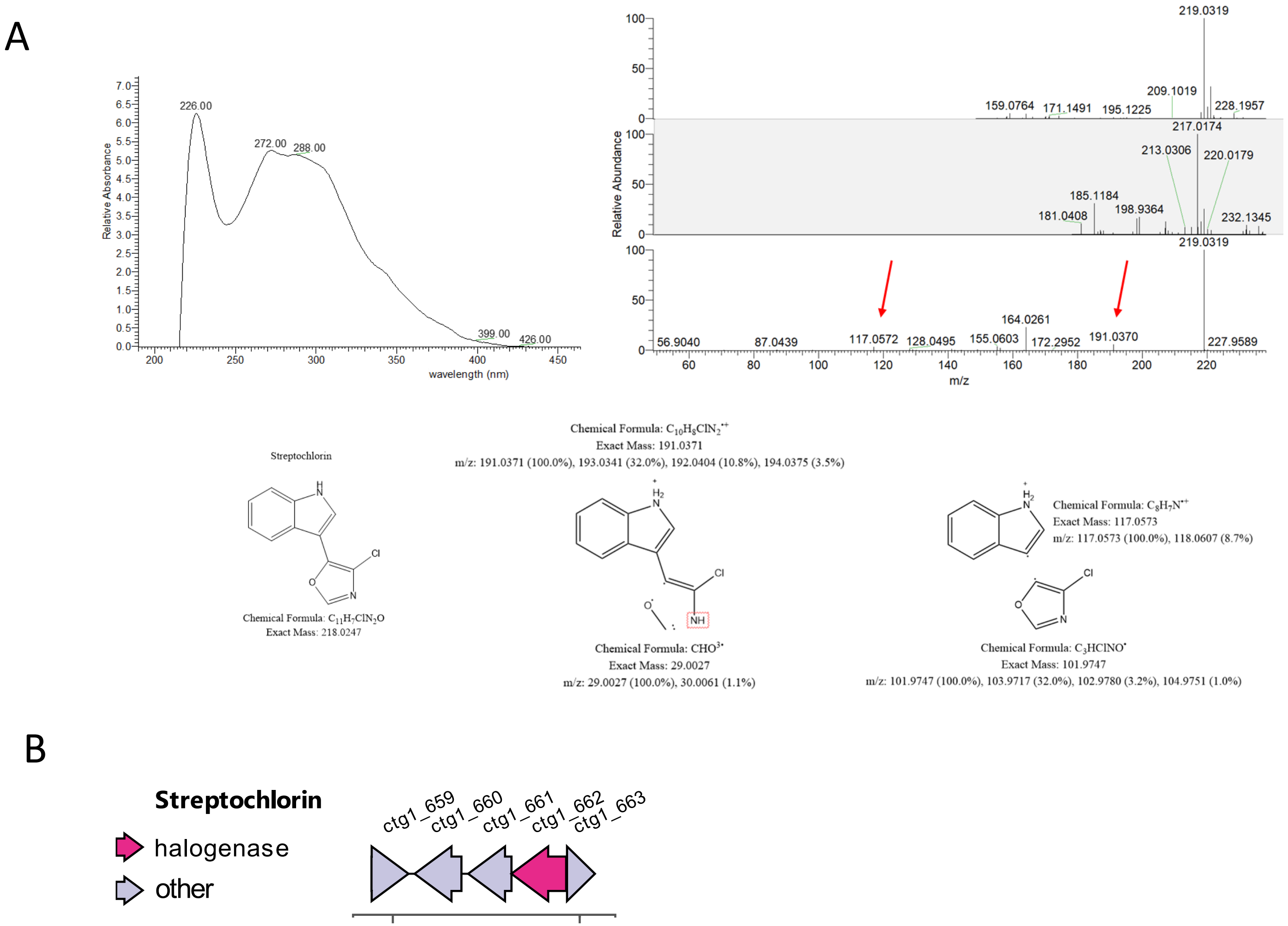

Figure S3. A) Identification of streptochlorin: top, left to right: UV spectrum of boxed area 3 in Fig. 2, MS spectra (from top to down): full mass in positive ionization mode, full mass in negative ionization mode and MS2 of *m/z* 219.0319 $[M+H]^+$, Bottom, structure of streptochlorin and predicted structures of MS fragments indicated by arrows. B) Predicted stretochlorine BGC from portion of Region 3 from Table 1. Genes are coloured as: pink, halogenase; grey, other biosynthetic functions. All genes are listed in Supplementary Table S2 (page S21).

A

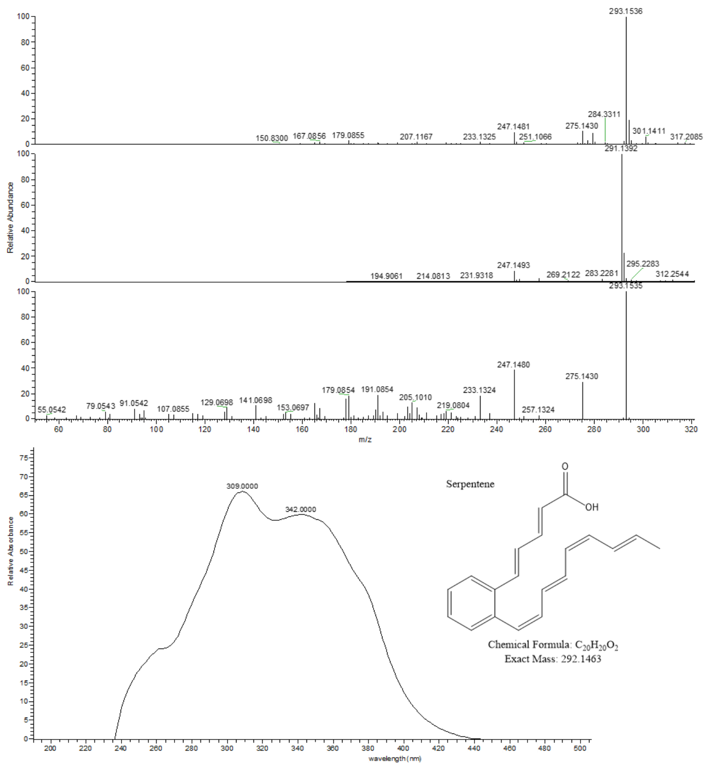

B **Serpentene**

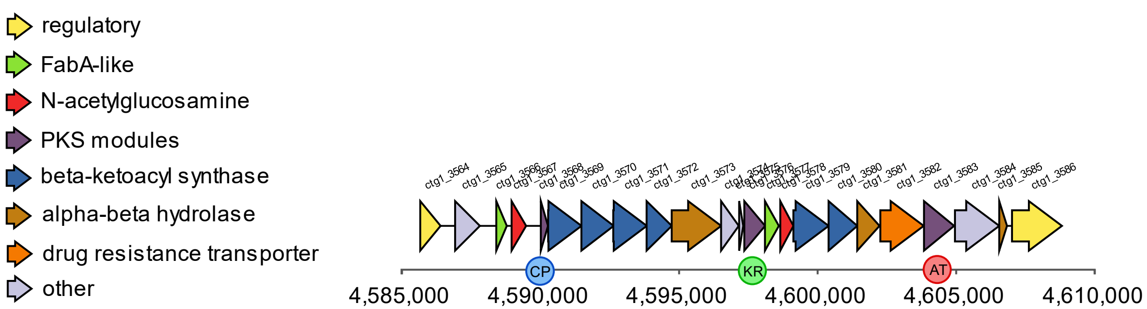

Figure S4. A) Identification of serpentene: top, MS spectra (from top to bottom): full mass in positive ionization mode, full mass in negative ionization mode and MS2 of *m/z* 293.1535 $[M+H]^+$, bottom, UV spectrum and serpentene structure. B) Predicted serpentene BGC from Region 18 of Table 1. All genes are listed in Supplementary Table S3 (page S22).

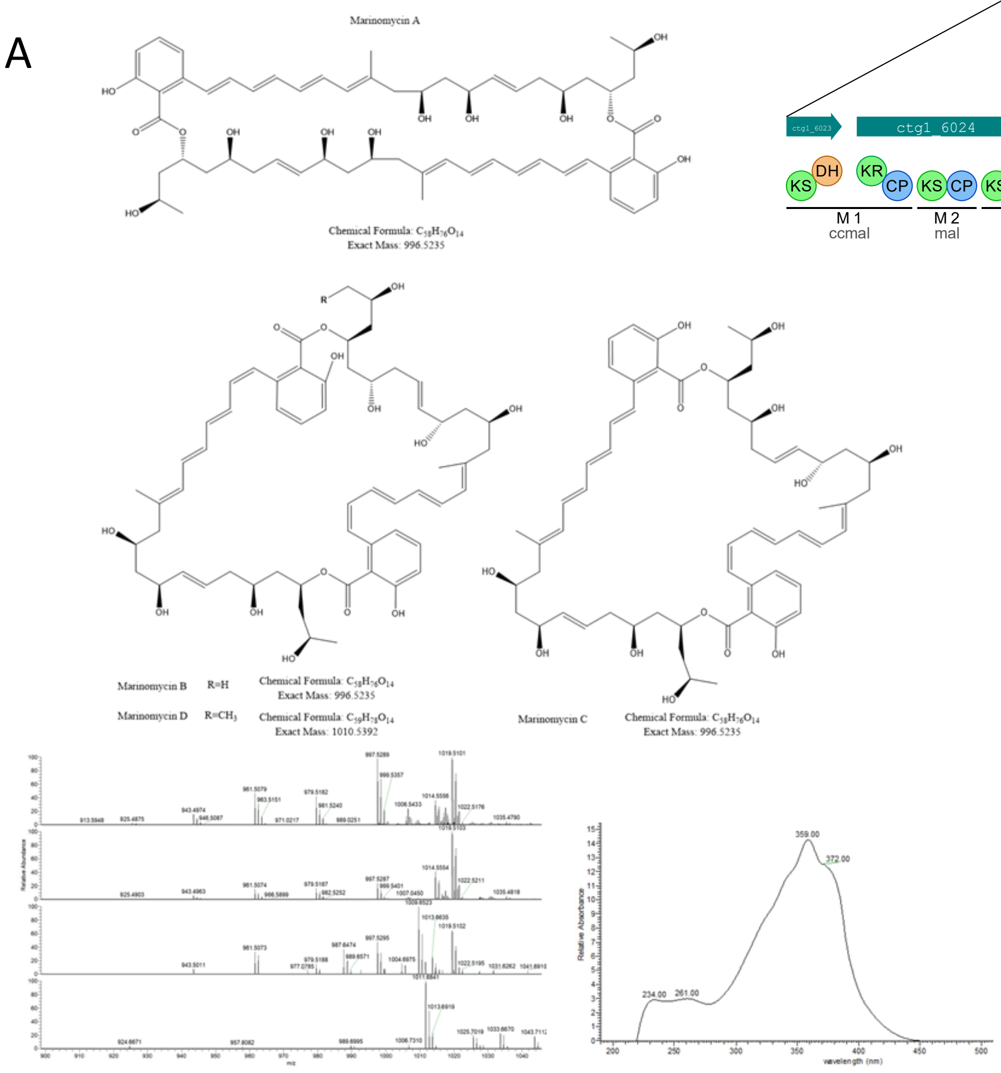

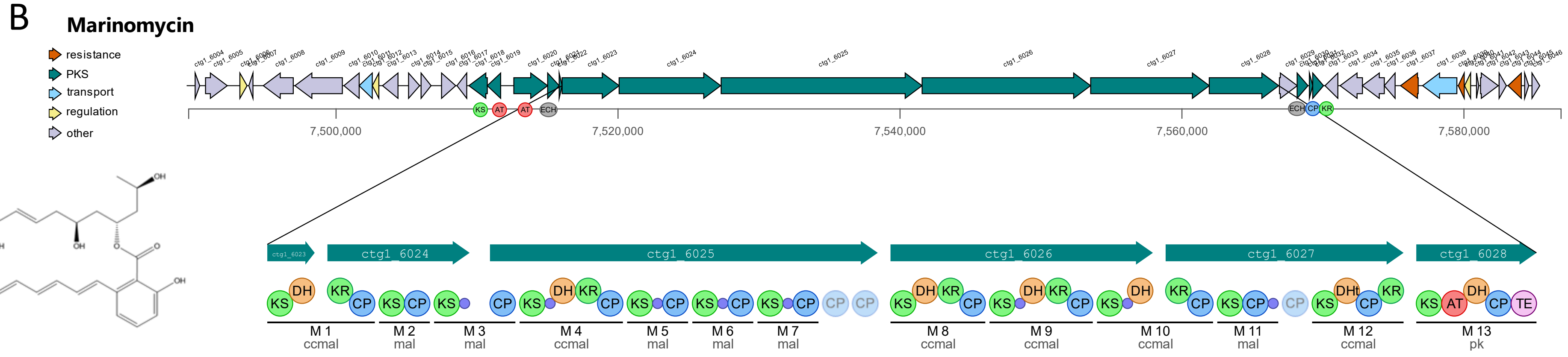

Figure S5. A) Identification of marinomycins: top, structures of identified congeners; bottom, HR full masses in positive ionization mode (left) and UV spectrum (right). Stereochemistry is assumed to be as reported in Natural Product Atlas (https://www.npatlas.org/). B) Predicted marinomycins BGC. Red arrows indicate genes that are a part of HMGS cassette. The enlargement shows the PKS domains. All genes are listed in Supplementary Table S4 (page S23).

A B

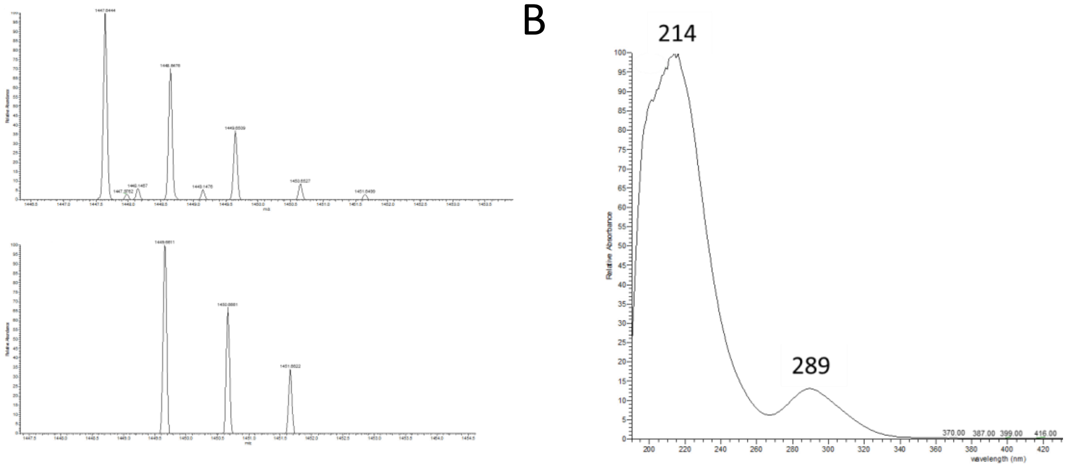

C

	1 found	1 calculated	2 found	2 calculated
$[M+H]^+$	1447.6494	1447.6448	1449.6614	1449.6605
$[M+2H]^{2+}$	724.3264	724.3260	725.3340	725.3339
y1			1313.6426	1313.6444
y2			1256.6239	1256.6224
b2			194.0445	194.0453
y3			1098.5533	1098.5543
y4			898.4380	898.4372
y5			797.3906	797.3895
b5			653.2780	653.2782
y6			660.3340	660.3306
b6	788.3239	788.3215	790.3378	790.3372
y7	561.2629	561.2627	561.2608	561.2622
b7	887.3901	887.3899	889.4062	889.4056
y8	433.2045	433.2041		
b8	1015.4484	1015.4485	1017.4633	1017.4641
y9	275.1351	275.1350		
b9	1173.5211	1173.5176	1175.5353	1175.5333
b10	1230.5375	1230.5391	1232.5507	1232.5548
b11	1317.5699	1317.5711	1319.5892	1319.5868

F

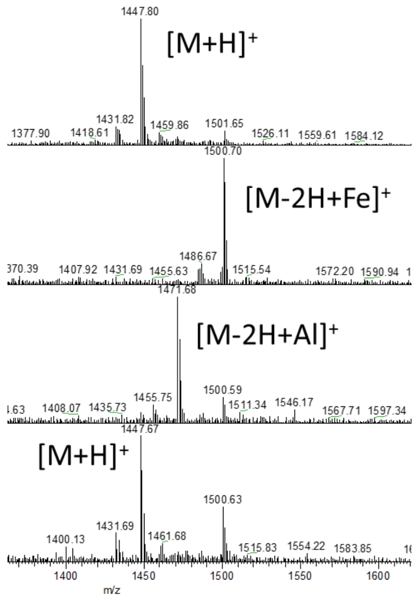

D

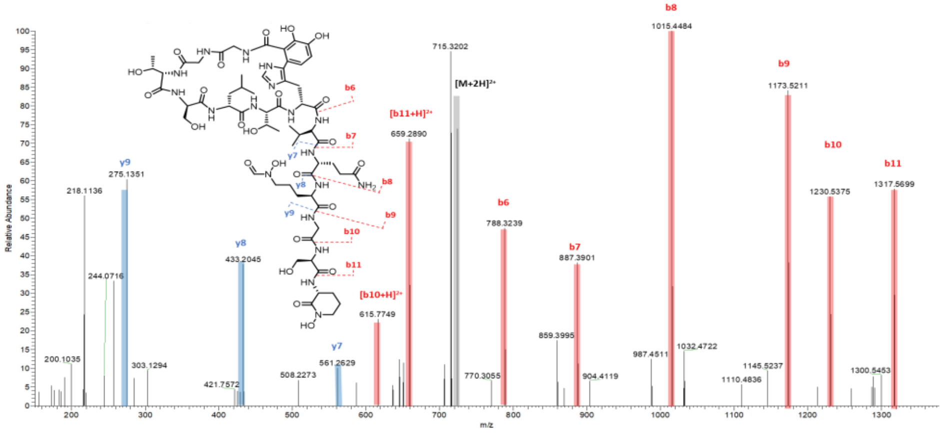

E

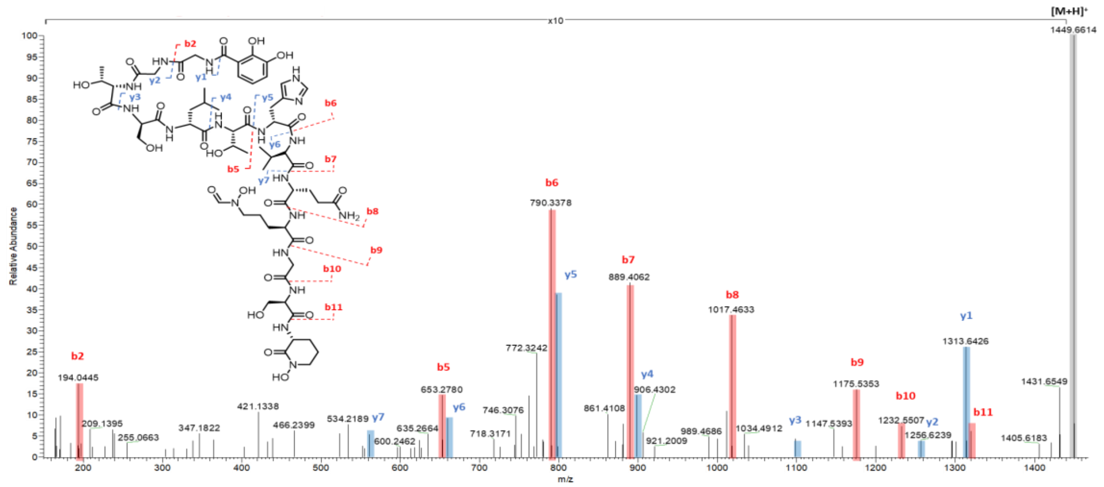

Figure S6. HR-LC-MS analysis of compounds **1** and **2**. A) MS spectra of **1** (top) and **2** (bottom); B) UV spectrum of **1**; C) assigned fragments for **1** and **2**, D-E) fragmentation spectra and assignment of fragments for **1** (panel D) and **2** (panel E); F) LR-MS spectra of **1**, **1** + $FeCl_3$, **1** + $AlCl_3$ and **1**+$ZnCl_2$.

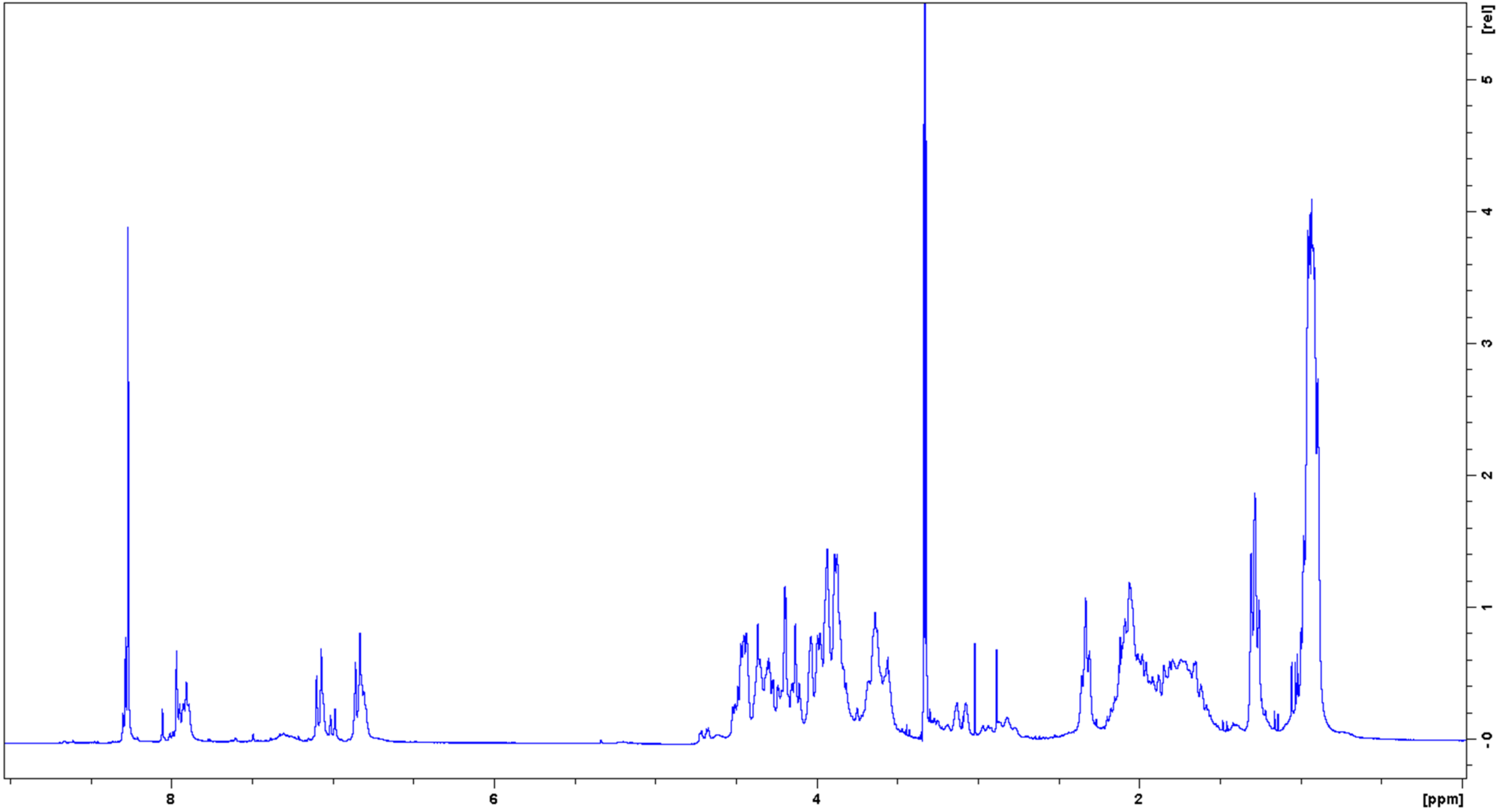

Figure S7. ^{1}H NMR (300 MHz, $CD_3OD:H_2O$ 9.8:0.2) of **1**.

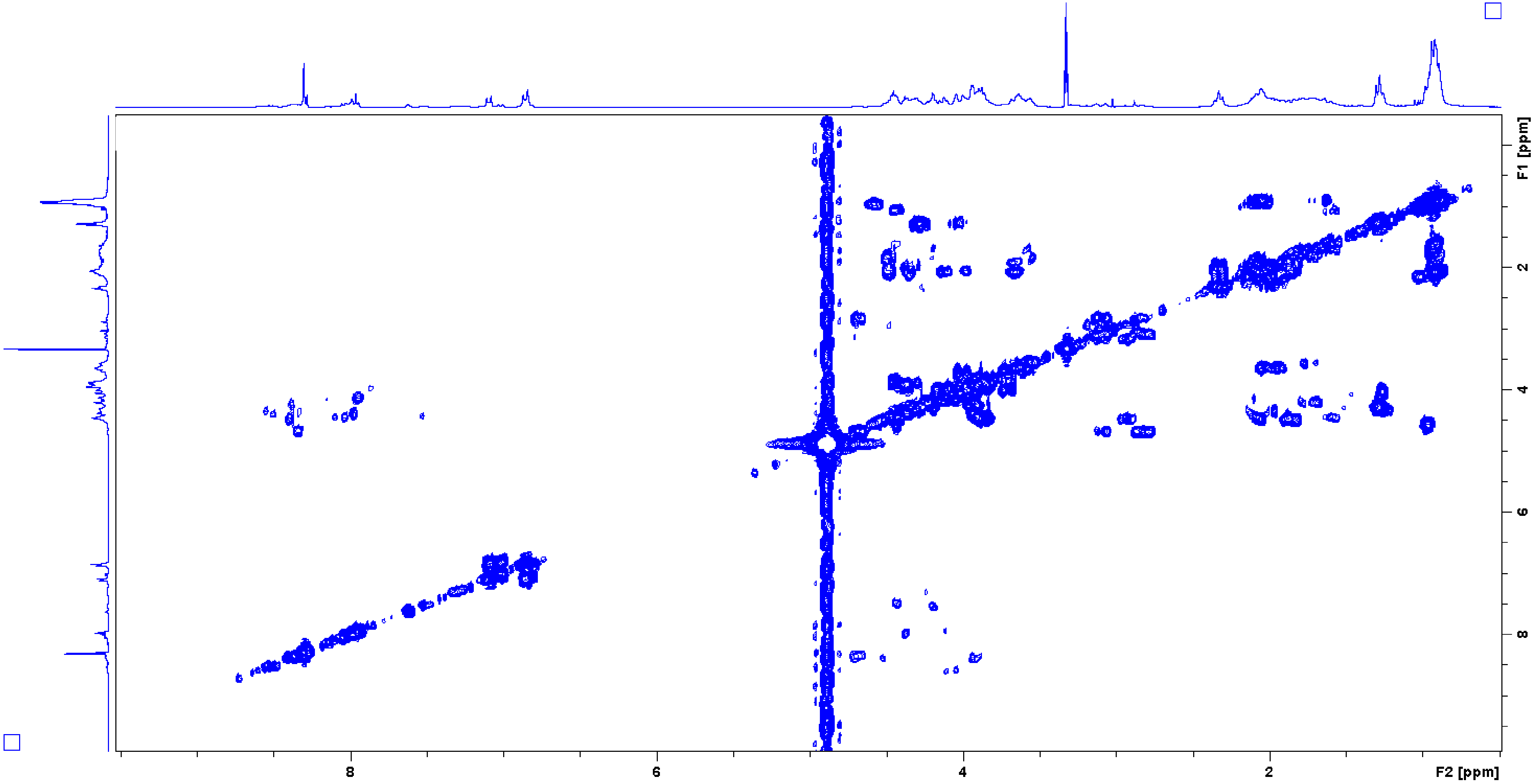

Figure S8. ^{1}H-COSY NMR (300 MHz, $CD_3OD:H_2O$ 9.8:0.2) of **1**.

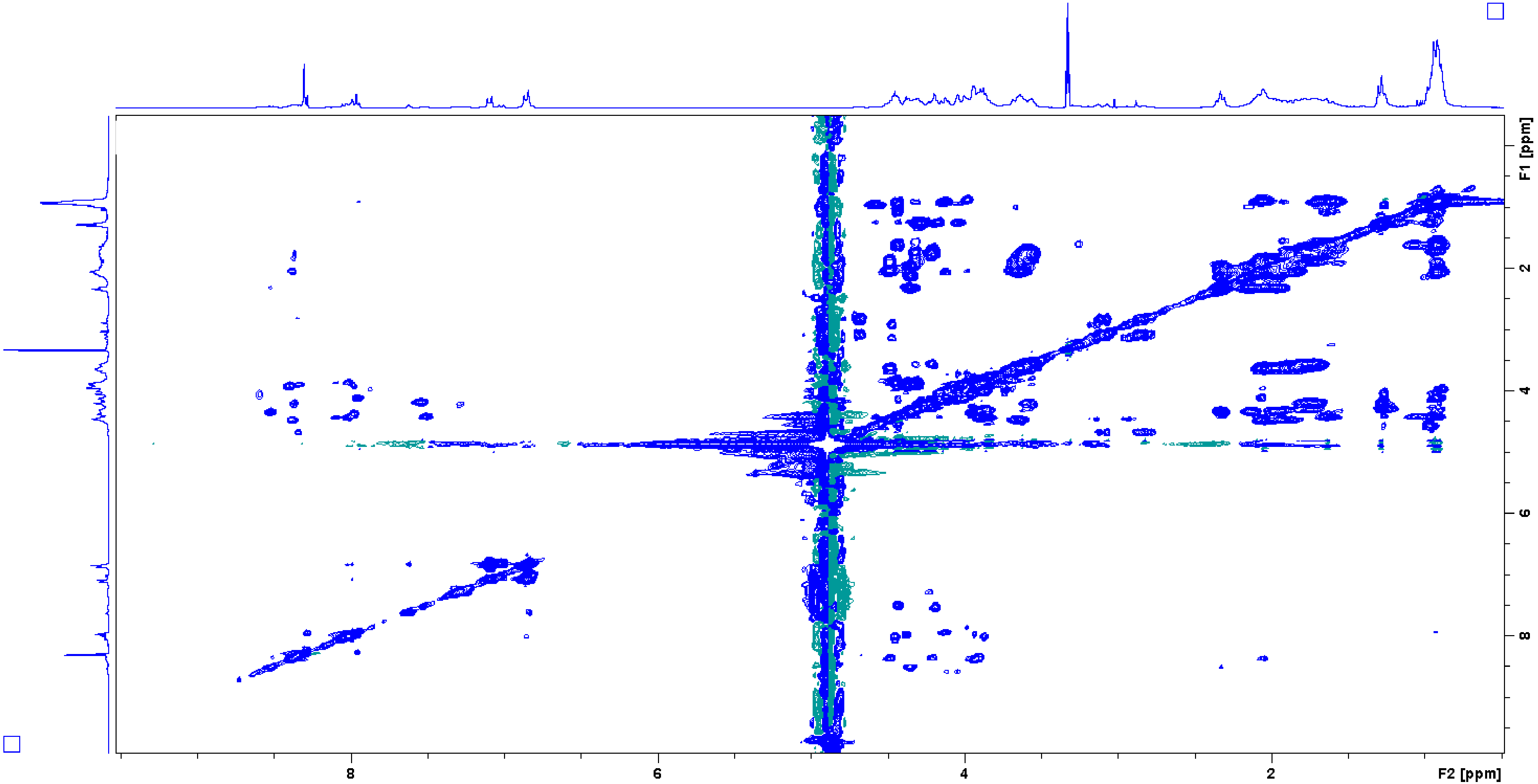

Figure S9. ^{1}H-TOCSY NMR (300 MHz, $CD_3OD:H_2O$ 9.8:0.2) of **1**.

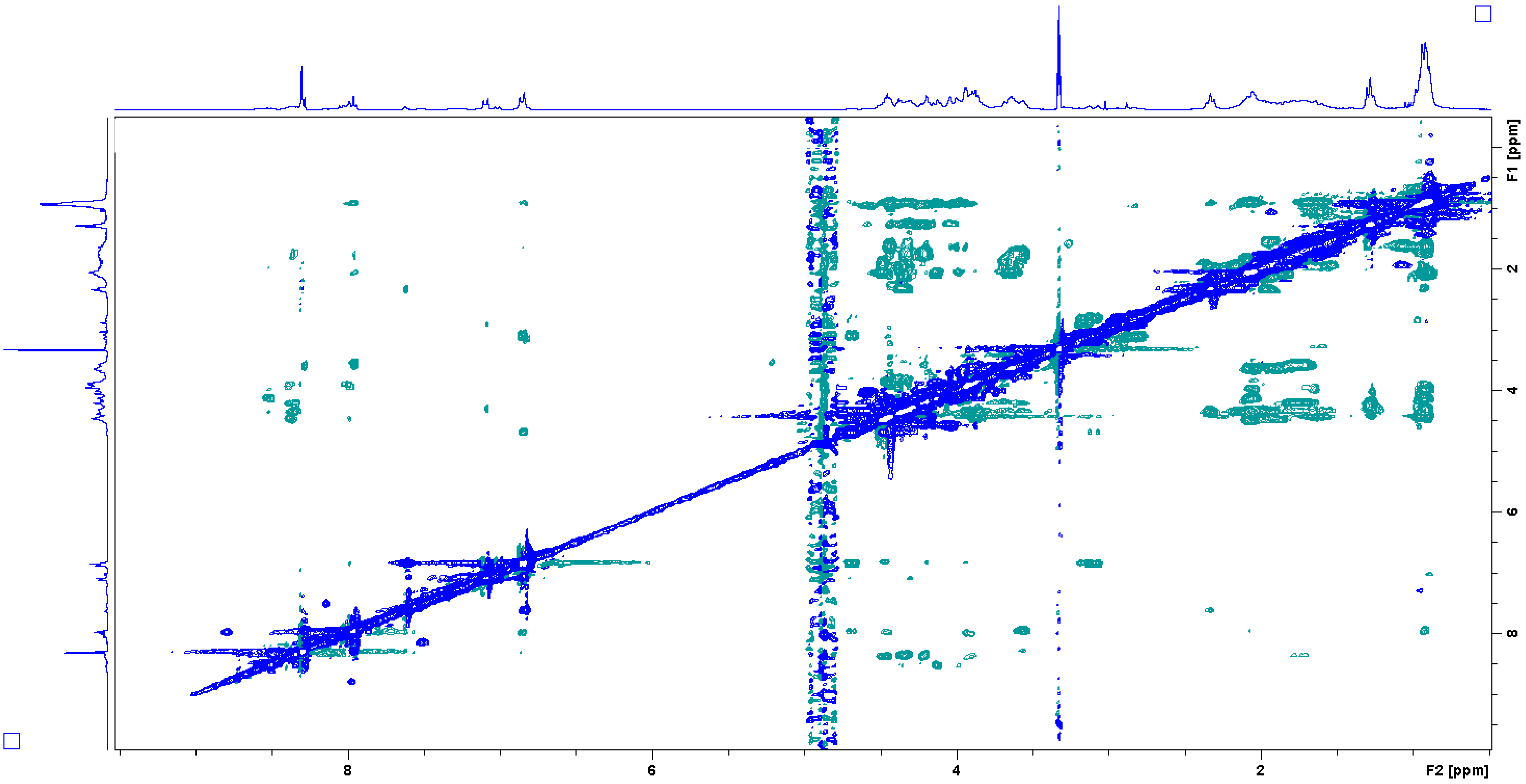

Figure S10. ^{1}H-ROESY NMR (300 MHz, $CD_3OD:H_2O$ 9.8:0.2) of **1**.

A

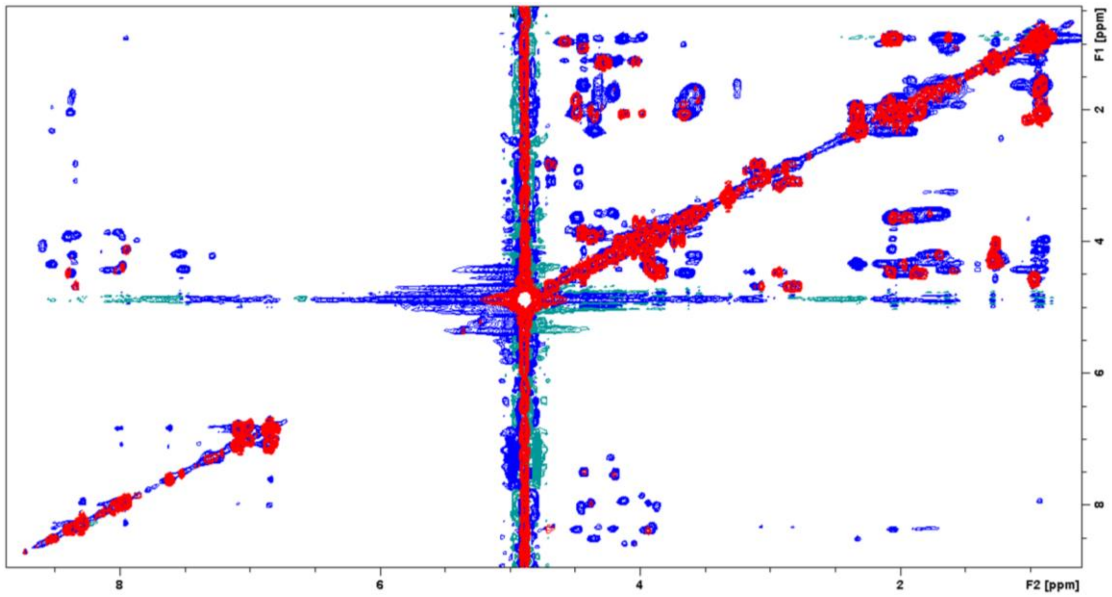

B

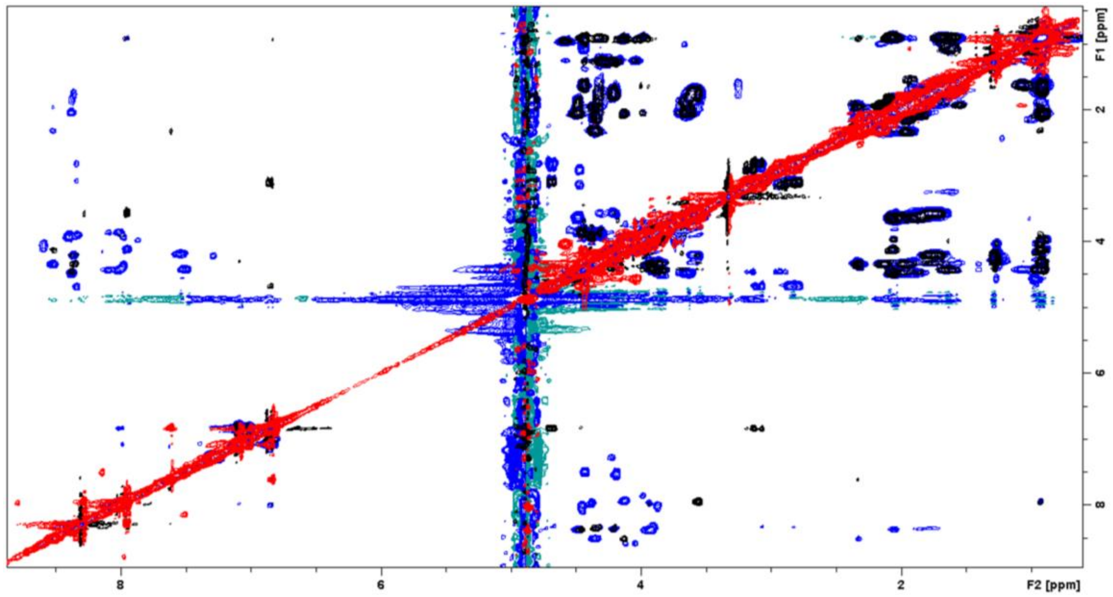

C

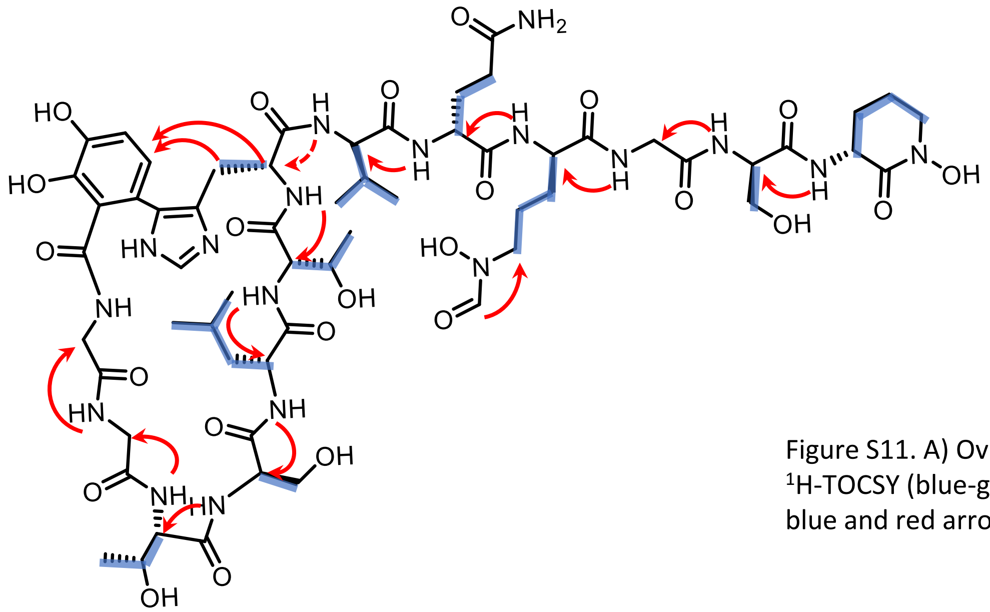

Figure S11. A) Overlap of ^{1}H-COSY (red) and ^{1}H-TOCSY (blue-green) NMR and B) overlap of ^{1}H-ROESY (red-black) and ^{1}H-TOCSY (blue-green) (300 MHz, $CD_3OD:H_2O$ 9.8:0.2) of **1**. C) Structure of **1** with COSY correlations highlighted in blue and red arrows showing main ROESY correlations.

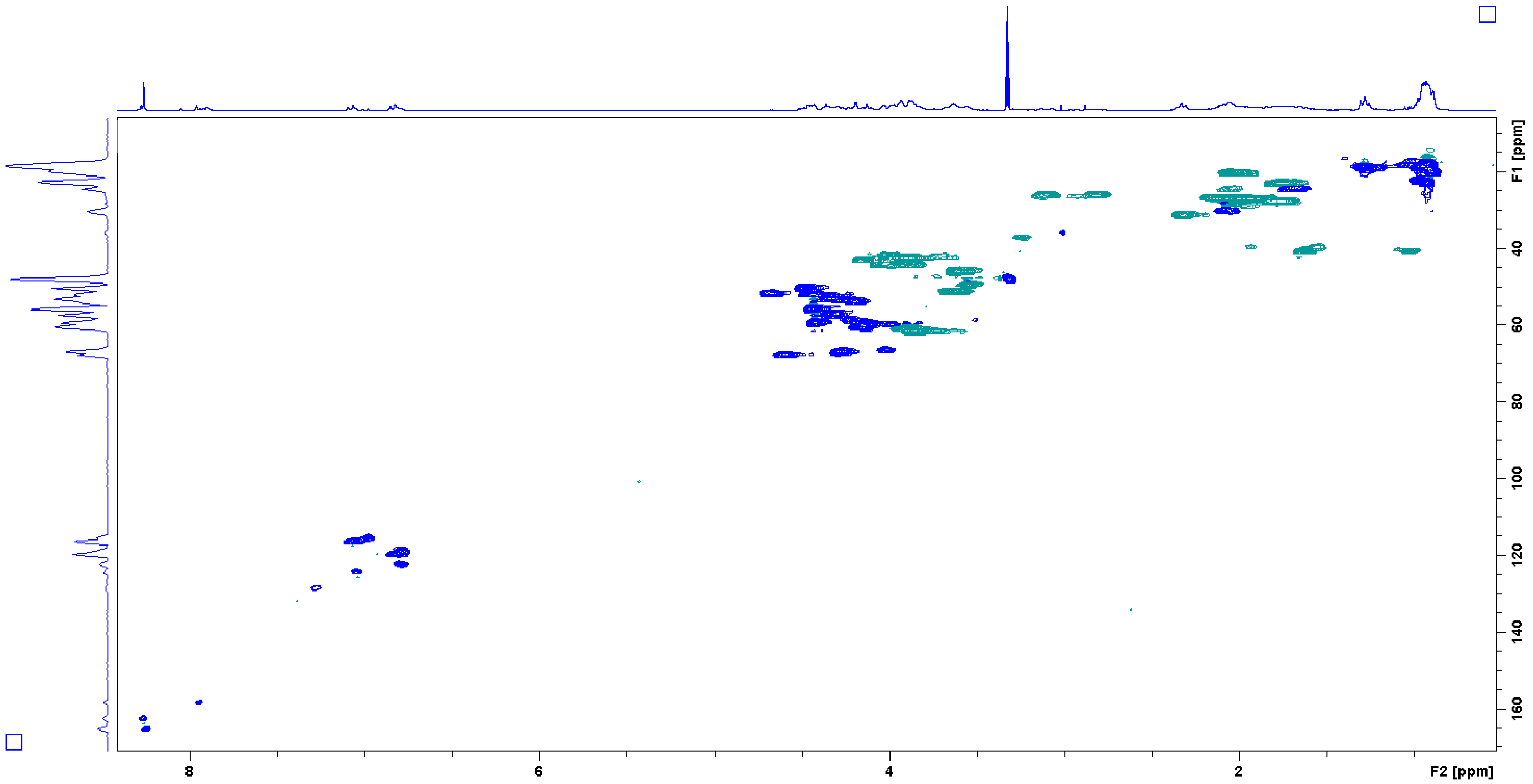

Figure S12. A) ^1H-^{13}C HSQC NMR (300 MHz, CD_3OD:H_2O 9.8:0.2) of **1**.

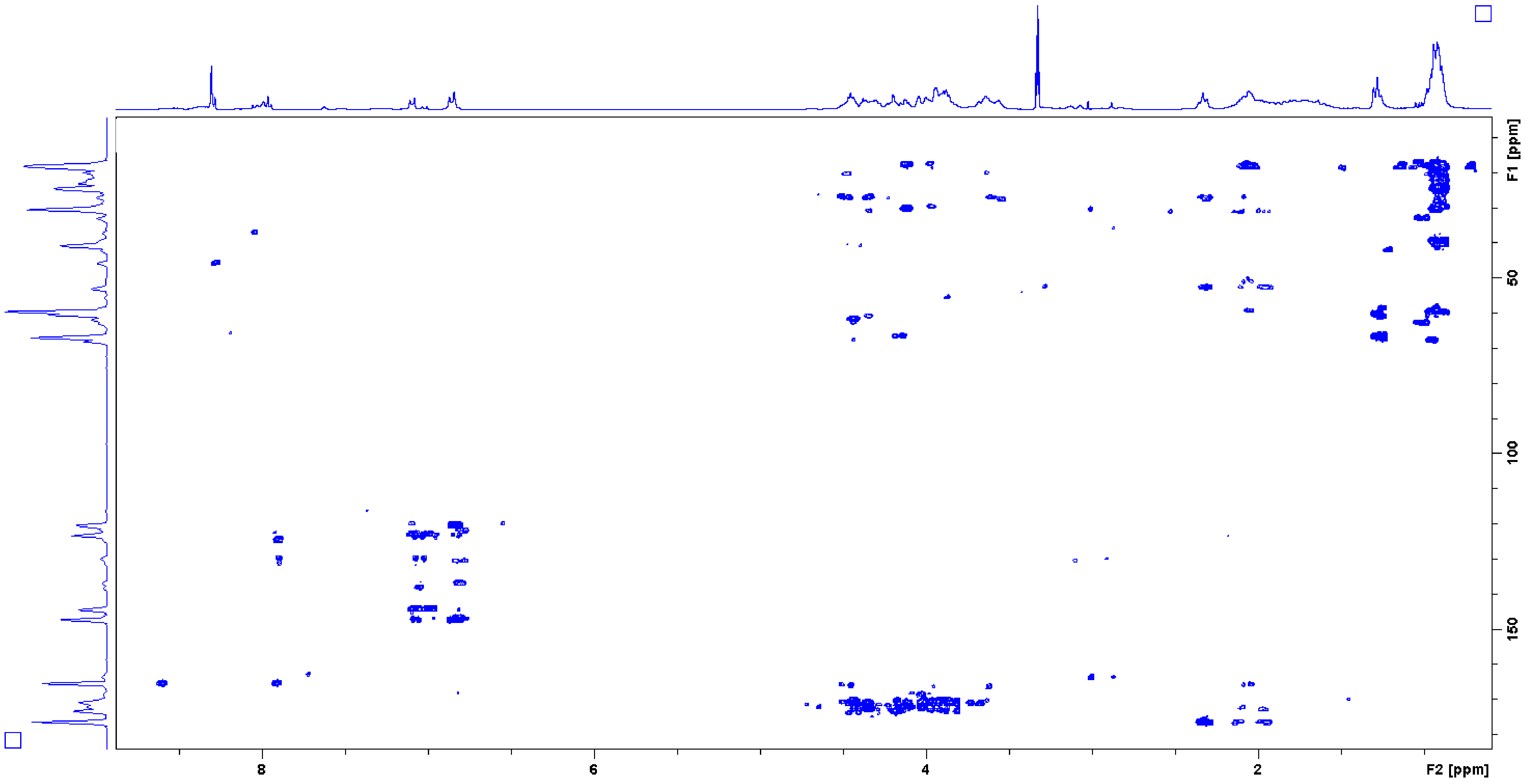

Figure S13. A) ^{1}H-^{13}C HMBC NMR (300 MHz, CD_3OD:H_2O 9.8:0.2) of **1**.

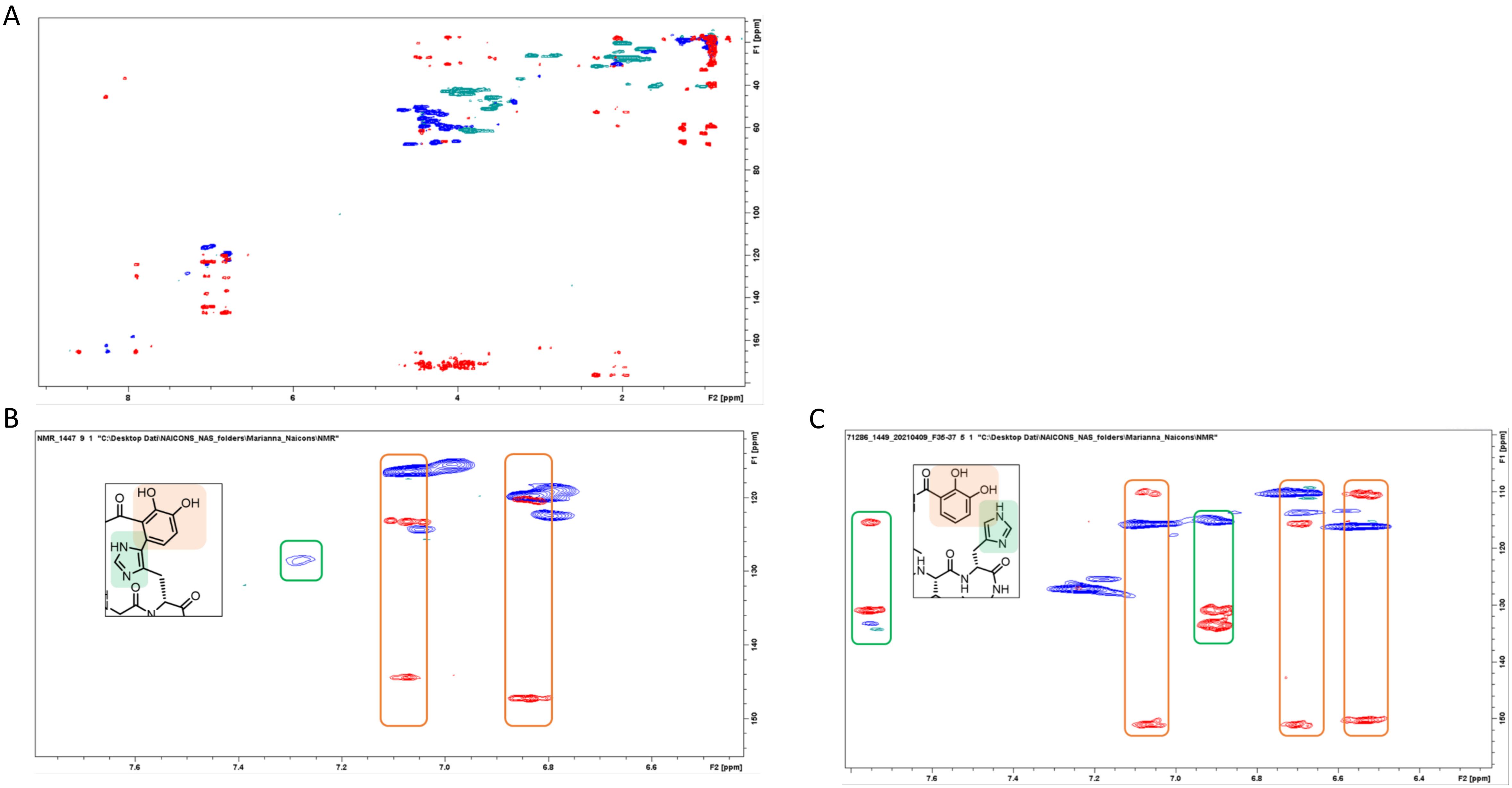

Figure S14. A) Overlap of ^{1}H-^{13}C HSQC (blue/green) and ^{1}H-^{13}C HMBC (red) (300 MHz, CD_3OD:H_2O 9.8:0.2) of **1**. B-C) selected HSQC-HMBC region of **1** (B) and **2** (C) (see manuscript for details).

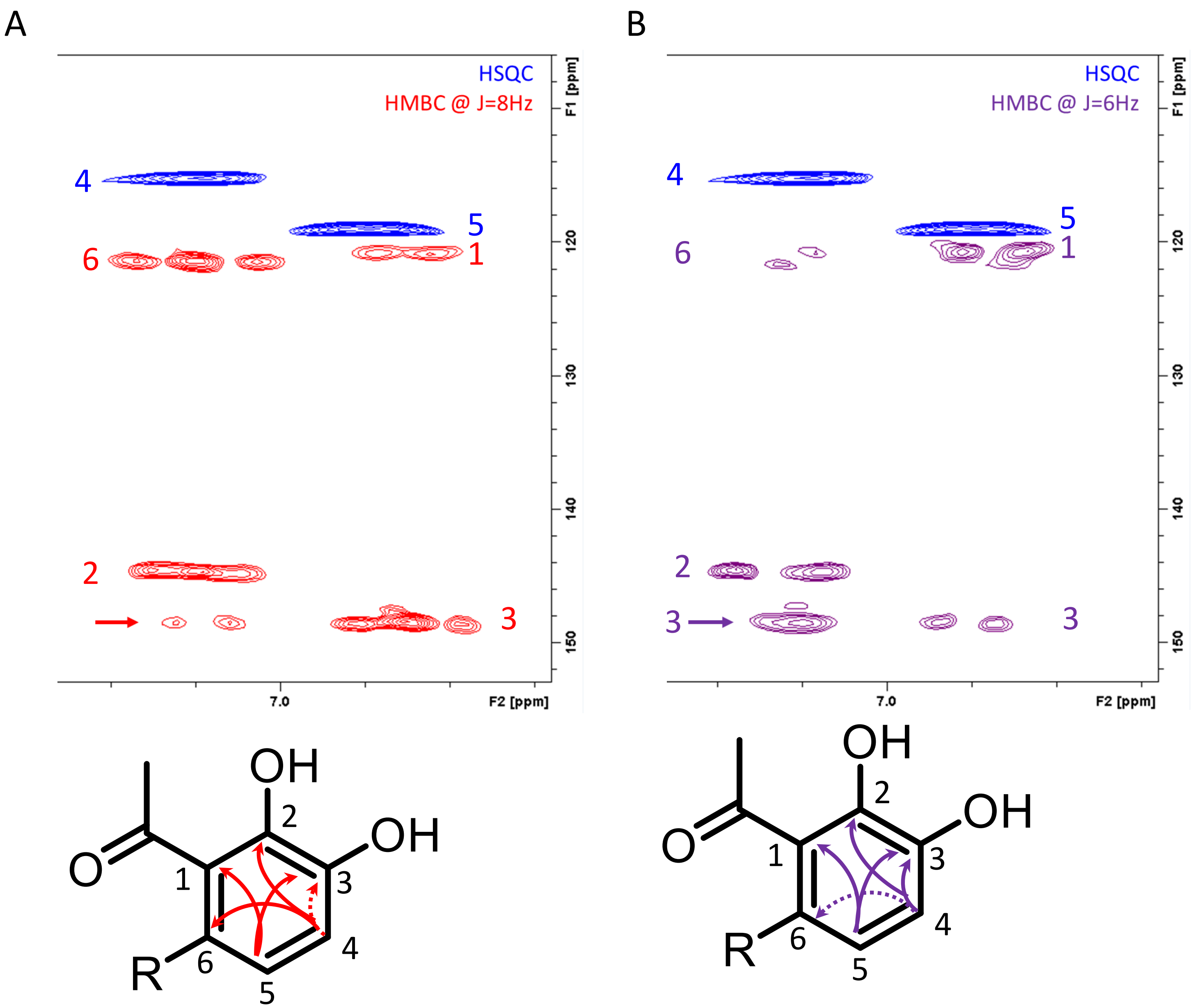

Figure S15. A) Selected region reporting an overlap of ^{1}H-^{13}C HSQC (blue) and ^{1}H-^{13}C HMBC optimised for JCH long-range couplings of 8 Hz (red) (300 MHz, $DMSO_{d6}$), B) selected region reporting an overlap of ^{1}H-^{13}C HSQC (blue) and ^{1}H-^{13}C HMBC optimised for JCH long- and short-range couplings of 6 Hz (purple) (300 MHz, $DMSO_{d6}$), and the corresponding assignments.

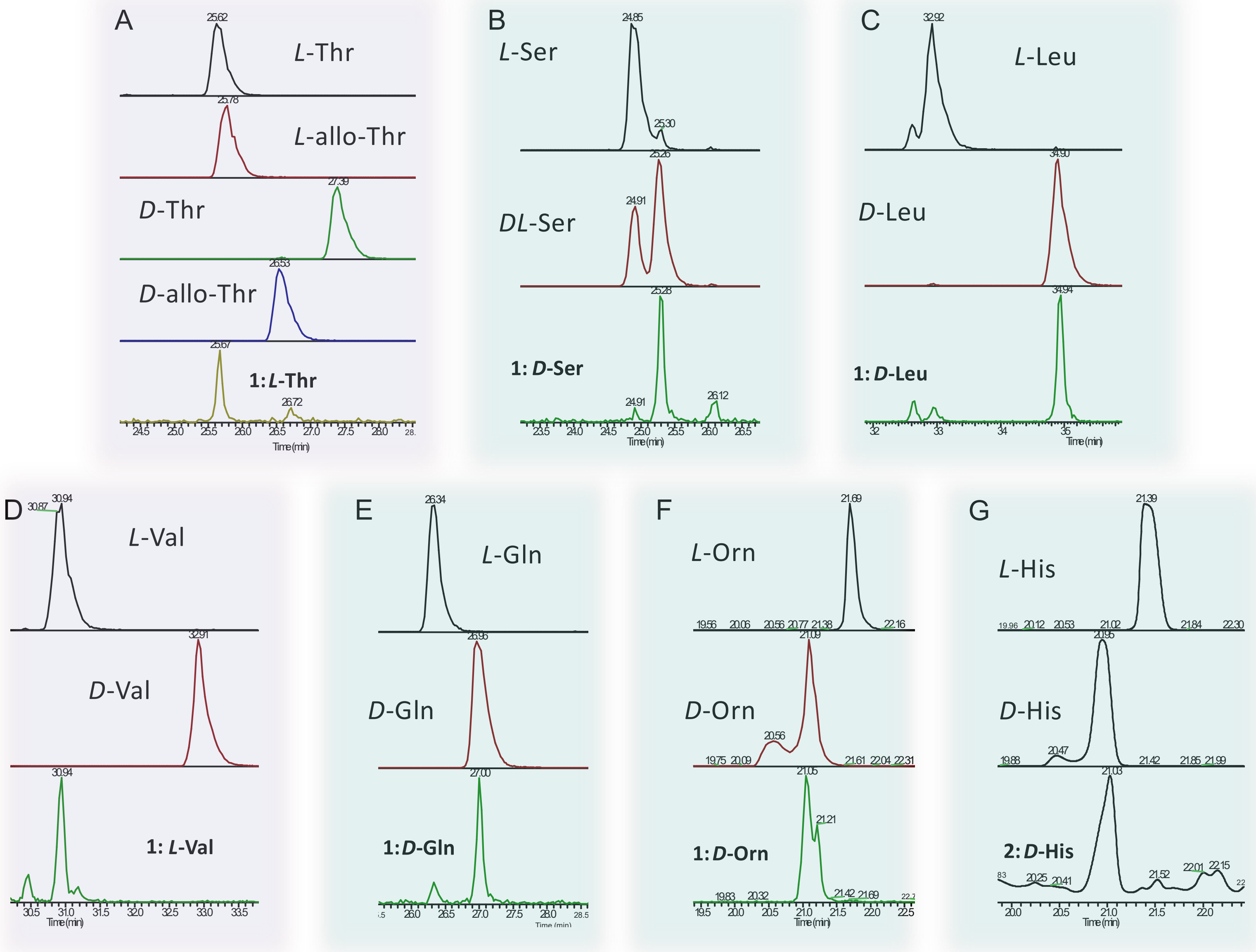

Figure S16. Extracted ion chromatograms of amino acid standards and compounds after treatment according to Marfey's method. Extracted ion chromatograms of threonine (A), serine (B), leucine (C), valine (D), glutamine (E), ornithine (F) and histidine standards (G) aligned with the hydrolysis products of **1** (A to F) and its linear form **2** (G).

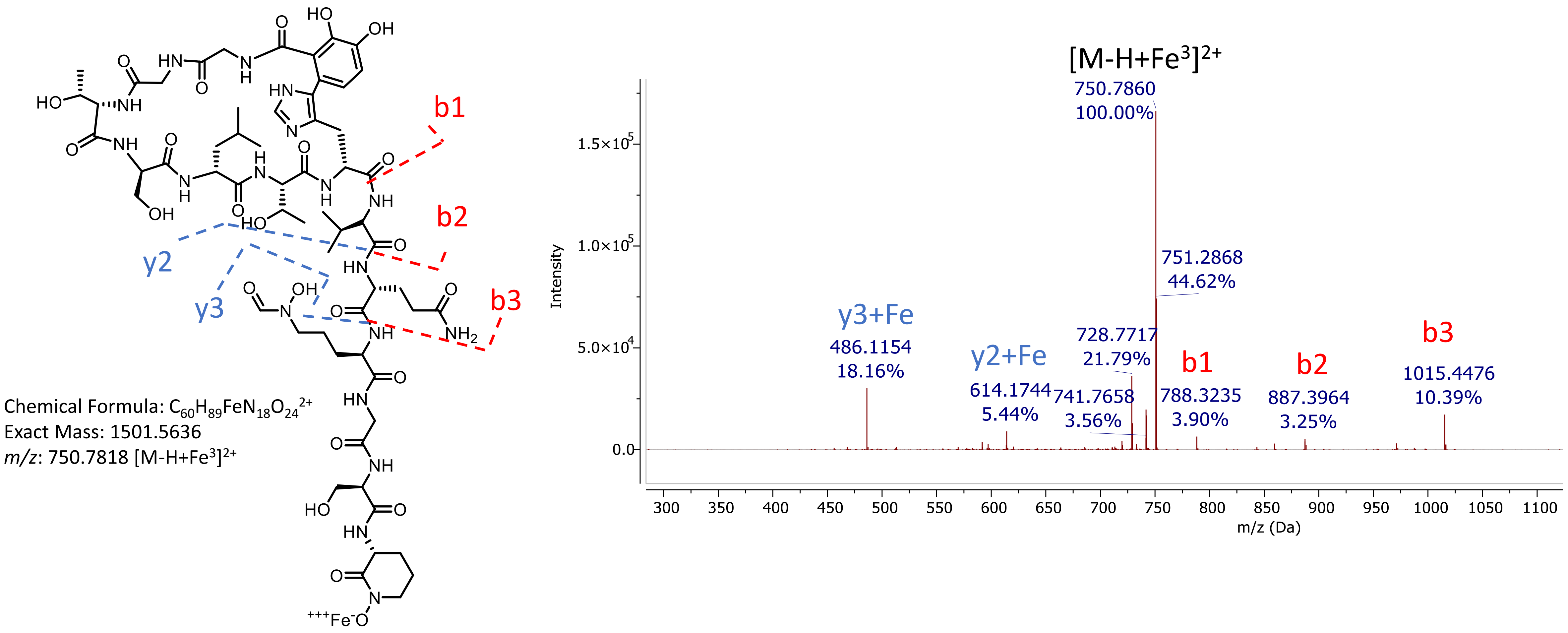

Figure S17. Iron-complexed **1**. Left: structure, chemical formula, calculated exact mass, calculated m/z of the double charged ion. Right: fragmentation pattern of m/z 750.7860^{2+} corresponding to $[M\text{-}H+Fe^3]^{2+}$, fragment assignment.

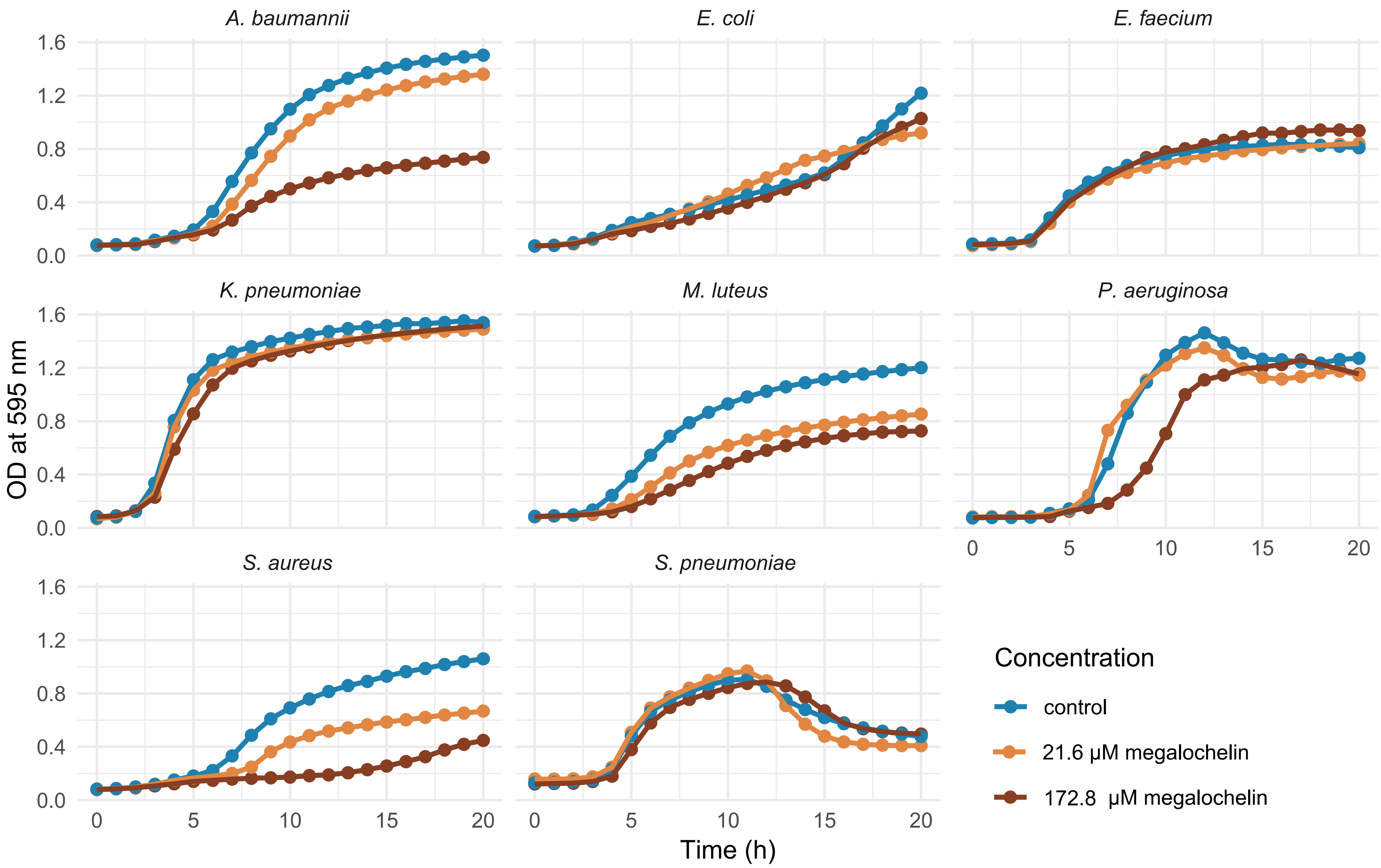

Figure S18. Growth curves of A. *baumannii, E. coli, E. faecium, K. pneumoniae, M. luteus, P. aeruginosa, S. aureus* and *S. pneumoniae* in the presence of megalochelin. The concentration of megalochelin is expressed in µM.

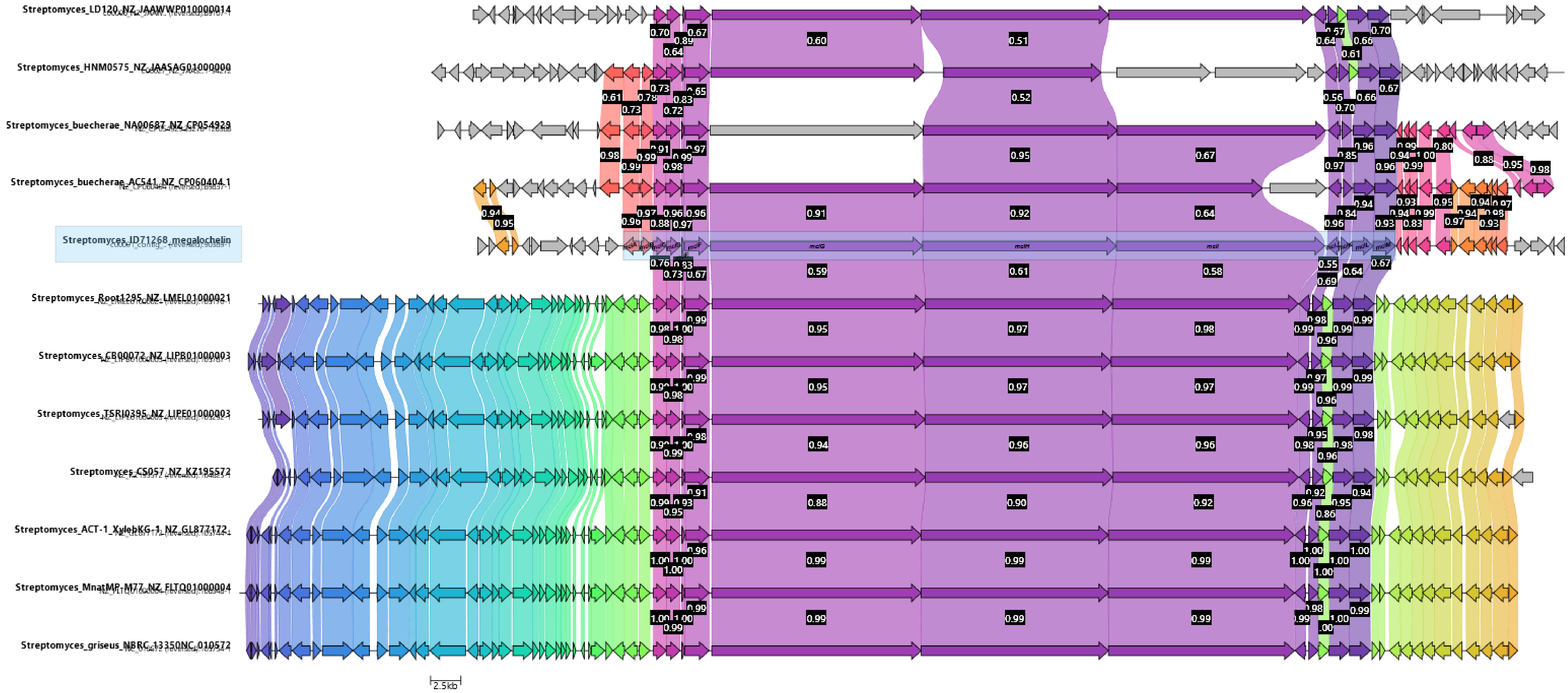

Figure S19. Comparison of megalochelin BGC comparison with similar BGCs identified in NCBI database by antiSMASH. Pairwise identity indicated between genes. Blue box defines the megalochelin BGC borders. Figure created with clinker.

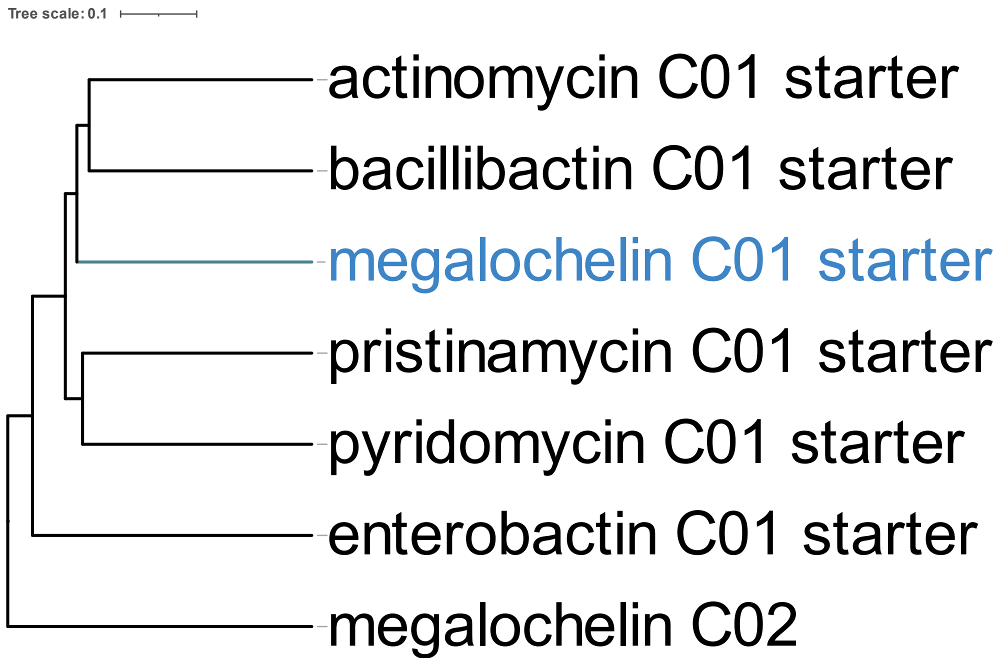

Figure S20. Amino acid sequence similarity tree comparing the starting C-domain of megalochelin BGC to starting C-domains of various metabolites incorporating DHB. Megalochelin second C-domain used as an outgroup.

Table S1. Genome properties of Streptomyces sp ID71268

Scaffolds	CG%	Accession number	Length bp	CDS
1	73%	BioProject ID PRJNA907813	8379354	8210

Table S2. Genes in streptochlorin BGC

Region 5

ID	Length (aa)	Predicted Function	smcogs/PFAM	E value	Score
ctg1_659	401	Glucose / Sorbosone dehydrogenase	PF07995.13	8.60E-65	219.3
ctg1_660	507	FAD binding domain	PF01565.25	1.10E-26	93.3
ctg1_661	469	sodium/hydrogen exchanger	SMCOG1117	2.00E-96	317.5
ctg1_662	594	Tryptophan halogenase	PF04820.16	2.30E-15	56.5
ctg1_663	302	no prediction			

Table S3. Genes in serpentene BGC

Region 18

ID	Length (aa)	Predicted Function	smcogs/PFAM	E value	Score
ctg1_3564	245	Archaeal transcriptional regulator TrmB	PF11495.10	0.000057	22.5
ctg1_3565	299	Recombinase	PF07508.15	7.00E-13	49
ctg1_3566	86	FabA-like domain	PF07977.15	5.20E-12	45.7
ctg1_3567	175	UDP-3-O-[3-hydroxymyristoyl] N-acetylglucosamine	SMCOG1270	4.30E-20	66.4
ctg1_3568	86	PP-binding domain	PF00550.27	6.50E-16	50.5
ctg1_3569	403	Beta-ketoacyl synthase	SMCOG1022	1.60E-99	327.9
ctg1_3570	390	Beta-ketoacyl synthase	SMCOG1022	6.60E-31	101.9
ctg1_3571	405	Beta-ketoacyl synthase	SMCOG1022	5.70E-58	191.1
ctg1_3572	304	Beta-ketoacyl synthase	PF00109.28	4.10E-12	46.3
ctg1_3573	598	Alpha/beta hydrolase family	PF12697.9	3E-09	37.9
ctg1_3574	213	DSBA-like thioredoxin domain	PF01323.22	2.20E-20	73.4
ctg1_3575	48	N/A			
ctg1_3576	250	inactive KR domain, enoyl-(Acyl carrier protein) reductase	SMCOG1001	1.80E-66	224.1
ctg1_3577	167	FabA-like domain	PF07977.15	2.9E-08	33.5
ctg1_3578	158	UDP-3-O-[3-hydroxymyristoyl] N-acetylglucosamine	SMCOG1270	5.40E-21	69.4
ctg1_3579	426	Beta-ketoacyl synthase	SMCOG1022	1.10E-88	292.3
ctg1_3580	127	Beta-ketoacyl synthase	PF00109.28	2.6E-06	27.3
ctg1_3581	269	Alpha/beta hydrolase family	PF12697.9	6.80E-11	43.3
ctg1_3582	524	Drug resistance transporter, EmrB/QacA	SMCOG1005	1.20E-123	407.2
ctg1_3583	364	malonyl CoA-acyl carrier protein transacylase	SMCOG1021	6.20E-91	300.1
ctg1_3584	535	carboxyl transferase	SMCOG1059	4.80E-219	722.6
ctg1_3585	93	Acyl-CoA carboxylase epsilon subunit	PF13822.8	5.90E-11	42.9
ctg1_3586	603	cetyl-CoA carboxylase biotin carboxylase	SMCOG1090	2.30E-206	681.5

Table S4. Genes in marinomycin BGC

Region 29a

ID	Length (aa)	Predicted Function	smcogs/PFAM	E value	Score
ctg1_6004	107	N/A			
ctg1_6005	513	Amino acid carrier protein	SMCOG1153	2.20E-125	413
ctg1_6006	169	transcriptional regulator, MerR family	SMCOG1171	1.60E-29	97.9
ctg1_6007	97	yiaA/B two helix domain	PF05360.16	6.20E-17	61.6
ctg1_6008	706	SMI1 / KNR4 family (SUKH-1)	PF09346.12	1.80E-08	34.9
ctg1_6009	1119	Peptidase_S8	PF00082.24	2.20E-64	216.2
ctg1_6010	359	N/A			
ctg1_6011	310	ABC transporter ATP-binding protein	SMCOG1000	6.10E-50	164.3
ctg1_6012	153	GntR family transcriptional regulator	SMCOG1136	9.40E-20	65.4
ctg1_6013	364	Endonuclease/Exonuclease/phosphatase family	PF03372.25	2.80E-08	33.7
ctg1_6014	261	Protein of unknown function (DUF4232)	PF14016.8	2.20E-27	95.9
ctg1_6015	236	RNase H	PF00075.26	2.80E-29	102.3
ctg1_6016	333	NAD-dependent epimerase/dehydratase	SMCOG1010	1.30E-26	88
ctg1_6017	230	N/A			
ctg1_6018	434	Beta-ketoacyl synthase	SMCOG1022	1.30E-96	318.4
ctg1_6019	316	PKS AT domain	PF00698.23	1.70E-27	96.9
ctg1_6020	798	PKS AT domain	PF00698.23	2.10E-37	129.4
ctg1_6021	272	ECH domain	SMCOG1023	1.20E-64	212.1
ctg1_6022	70	N/A			
ctg1_6023	1348	PKS			
ctg1_6024	2398	PKS			
ctg1_6025	4745	PKS			
ctg1_6026	3979	PKS			
ctg1_6027	2802	PKS			
ctg1_6028	1640	PKS			
ctg1_6029	421	hydroxymethylglutaryl-CoA synthase	SMCOG1043	4.90E-137	451.2
ctg1_6030	255	enoyl-CoA hydratase	SMCOG1023	3.10E-64	210.8
ctg1_6031	80	PP-binding domain	PF00550.27	1.40E-11	36.6
ctg1_6032	255	short-chain dehydrogenase/reductase SDR	SMCOG1001	4.80E-76	249.7
ctg1_6033	303	Universal stress protein family	PF00582.28	3.70E-16	60
ctg1_6034	526	Peptidase_S41	PF03572.20	2.50E-17	63.1
ctg1_6035	517	Amidohydrolase family	PF01979.22	5.50E-06	26
ctg1_6036	255	N/A			
ctg1_6037	416	beta-lactamase	SMCOG1053	5.70E-81	266.2
ctg1_6038	808	ABC transporter ATP-binding protein	SMCOG1000	2.20E-19	64.4
ctg1_6039	139	Glyoxalase/Bleomycin resistance protein/Dioxygenase superfamily	PF00903.27	3.00E-15	56.6
ctg1_6040	143	AraC family transcriptional regulator	SMCOG1287	1.50E-25	84.2
ctg1_6041	51	N/A			
ctg1_6042	418	N/A			
ctg1_6043	170	N/A			
ctg1_6044	312	Glyoxalase/Bleomycin resistance protein/Dioxygenase superfamily	PF00903.27	4.20E-15	56.2
ctg1_6045	96	N/A			
ctg1_6046	152	THAP4-like, heme-binding beta-barrel domain	PF08768.13	4.60E-46	156.6

Table S5. Predicted Functions of Proteins Present in the Megalochelin Biosynthetic Gene Cluster of Streptomyces ID 71268

Mcl	Length (aa)	Predicted Function	smcogs/PFAM	E value	Score
A	440	lysine N(6)-hydroxylase/L-ornithine N(5)-oxygenase family protein	SMCOG1080	9.50E-162	532.7
B	318	methionyl-tRNA formyltransferase	SMCOG1228	1.50E-131	432.2
C	365	iron chelate uptake ABC transporter family permease subunit	SMCOG1011	3.10E-100	329.5
D	384	iron chelate uptake ABC transporter family permease subunit	SMCOG1011	3.60E-86	283.3
E	72	MbtH family protein (DmdR1 binding site on gene)	SMCOG1009	1.20E-34	112.1
F	668	multicopper oxidase domain-containing protein	PF07732.16	1.90E-18	66.6
G	5883	non-ribosomal peptide synthetase			
H	5376	non-ribosomal peptide synthetase			
I	5768	non-ribosomal peptide synthetase			
J	331	ABC transporter substrate-binding protein	SMCOG1033	5.20E-57	187.3
K	267	vibriobactin utilization protein ViuB (DmdR1 binding site on gene)	SMCOG1200	1.10E-76	251.7
L	579	ABC transporter ATP-binding protein/permease	SMCOG1288	2.50E-131	433.2
M	612	ABC transporter ATP-binding protein/permease	SMCOG1288	7.30E-86	283.2

Table S6. Genes in BGC coding for the DHB starter unit of Megalochelin

Region 26					
ID	Length (aa)	Predicted Function	smcogs/PFAM	E value	Score
ctg1_5585	335	iron compound ABC transporter, periplasmic	SMCOG1033	2.9E-76	250.6
ctg1_5586	259	short-chain dehydrogenase/reductase SDR	SMCOG1001	1.9E-80	264.1
ctg1_5587	461	isochorismate synthase	SMCOG1018	6.8E-121	398.7
ctg1_5588	572	AMP-dependent synthetase and ligase	SMCOG1002	6.4E-152	500.6
ctg1_5589	210	isochorismatase	SMCOG1027	5.9E-93	305
ctg1_5590	76	PP-binding	PF00550.27	8.90E-14	51.7
ctg1_5591	1451	NRPS	SMCOG1127	2.1E-101	334.4
ctg1_5592	79	n/a			
ctg1_5593	198	GCN5-related N-acetyltransferase	SMCOG1111	1.3E-33	110.6
ctg1_5594	278	alpha/beta hydrolase fold protein	SMCOG1036	6.5E-16	53
ctg1_5595	371	transcriptional regulator, MerR family	SMCOG1171	3.2E-32	106.6
